# Development and internal validation of an interpretable machine learning model to predict coagulopathy following extracorporeal membrane oxygenation: a retrospective multicenter study

**DOI:** 10.1186/s13049-026-01564-x

**Published:** 2026-01-28

**Authors:** Zhen Chen, Zhenhua Zeng, Genglong Liu, Yongpeng Su, Changzhi Liu, Yiqi Zhong, Jiamin Li, Liuer Zuo

**Affiliations:** 1 Department of Intensive Care Unit, The Eighth Affiliated Hospital, Southern Medical University (The First People’s Hospital of Shunde, Foshan), Guangdong Province Foshan, 528308 People’s Republic of China; 2 Department of Critical Care Medicine, Nanfang Hospital, Southern Medical University, Guangdong Province Guangzhou, 510515 People’s Republic of China; 3 School of Medicine, Southern Medical University, Guangdong Province Foshan, 528305 People’s Republic of China; 4 Editor Office, iMeta, Guangdong Province Shenzhen, 518000 People’s Republic of China

**Keywords:** Extracorporeal membrane oxygenation, Coagulopathy, Prediction model, Machine learning approaches, Model interpretability

## Abstract

**Background:**

Extracorporeal membrane oxygenation-induced coagulopathy (ECMO-IC) represents a frequent and severe complication, contributing to oxygenator replacement and unfavorable outcomes. Currently, no reliable machine learning (ML) model exists for early identification. This study comprehensively assesses routine clinical characteristics to develop a reliable, accurate, and explainable ML model for estimating ECMO-IC risk and to identify modifiable factors.

**Methods:**

This study included two center cohorts with 266 patients undergoing ECMO from 2015 to 2024. Feature selection utilized the Boruta algorithm, followed by the implementation of a distinctive ML framework incorporating 12 ML algorithms to establish a consensus prediction model (ECMO-IC index). Model and feature variable assessment employed multiple analytical methods: Bootstrapping and fivefold cross-validation, subgroup and interaction analysis, restricted cubic spline (RCS) regression, and threshold effect analysis. Model interpretation and feature quantification relied on the Shapley Additive Explanations (SHAP) methodology for visualization purposes.

**Results:**

Through Boruta algorithm selection, 17 characteristics were identified and incorporated into 12 ML methodologies, generating 105 permutations and an optimal algorithm for identifying ECMO-IC. The ECMO-IC index comprising 9 modifiable or nonmodifiable variables, namely platelet (PLT), lactate, systemic immune-inflammation index (SII), K, total protein (TP), shock index (SI), red blood cell volume distribution width (RDWCV), acute physiology and chronic health evaluation II (APACHE II), and Ca, demonstrated strong diagnostic capabilities, achieving a mean area under the curve (AUC) of 0.815 across derivation (AUC = 0.817) and validation (AUC = 0.813) cohorts, along with notable discriminatory power, model fit, and clinical utility. SHAP elucidates the importance of ranking features (PLT, lactate, K, Ca and APACHE II) and visualises global and individual ECMO-IC risk prediction. RCS regression and threshold effect analysis suggested a nonlinear link between model features (PLT: *P* for nonlinearity = 0.002, SII: *P* for nonlinearity = 0.001, K: *P* for nonlinearity = 0.006, Ca: *P* for nonlinearity = 0.008, and lactate: *P* for nonlinearity = 0.004) and ECMO-IC, and generated an inflection point for features (PLT = 95 × 10^9^/L, lactate = 5.7 mmol/L, SII = 200, K = 4.4 mmol/L, TP = 45.6 g/L, SI = 0.8, RDWCV = 14%, APACHE II = 15, Ca = 1.03 mmol/L). To provide a more flexible predictive tool, the ECMO-IC model was constructed using a free, publicly available web-based calculator (https://genglongliu.shinyapps.io/DynNomapp/).

**Conclusion:**

An optimised explainable ML model (ECMO-IC index) incorporating several modifiable parameters was established and internally validated to deliver an readily available and accurate diagnostic tool for ECMO-IC, with potential applications in ECMO clinical management.

**Supplementary Information:**

The online version contains supplementary material available at 10.1186/s13049-026-01564-x.

## Introduction

Extracorporeal membrane oxygenation (ECMO) serves as a last-resort therapy to maintain organ perfusion and oxygen delivery in patients experiencing severe, reversible respiratory or circulatory dysfunction following the failure of conventional treatments [1]. In recent years, global ECMO utilization has experienced significant growth during the COVID-19 pandemic, demonstrating its effectiveness in resuscitating both pediatric and adult patients in critical conditions [2]. Despite advances in ECMO technology, the mortality rate among patients under ECMO support remains high, with an adult mortality rate of approximately 60% [3, 4]. This can be attributed to several contributing factors, such as ECMO-related complications, timing of ECMO therapy and selection indications, determining the appropriate timing for ECMO weaning and post-weaning management, and a specialised ECMO medical team, which are key elements and priorities for future research [4].

During ECMO, the contact between the blood and synthetic surfaces within the circuit may initiate acute inflammatory and prothrombotic reactions [5]. A frequent and acute complication in ECMO therapy is coagulopathy, an acquired, potentially fatal intravascular condition marked by activated systemic coagulation, disrupted fibrinolysis and damaged endothelium that eventually presents both bleeding and thrombotic complications [6]. On the one hand, ECMO-induced coagulopathy (ECMO-IC) not only increases flow resistance and equipment malfunction but also decreases the oxygenation processes, often requiring oxygenator replacement [7]. On the other hand, coagulopathy in ECMO patients resulted in an elevated mortality rate compared with non-coagulopathic patients [8]. Moreover, for ECMO-IC treatment, clinicians are required to maintain a delicate balance between anticoagulation and coagulation, and the simultaneous administration of supportive therapies such as blood products is also essential [9]. As the preparation and administration of fresh frozen plasma and platelet transfusions can be time-consuming, the ability to predict coagulopathy risk in patients receiving ECMO in advance would provide a significant benefit in clinical practice by enabling resource planning and timely intervention [10]. Thus, there is a pressing need to identify crucial and modifiable factors to mitigate ECMO-IC risk and to develop a noninvasive, reliable, and accessible evaluation model for early ECMO-IC identification.

The critical illness and complex physiology of patients receiving ECMO, along with the configuration of ECMO support parameters, make early diagnosis of ECMO-IC in adults particularly challenging. Machine learning (ML) has the potential to identify modifiable risk factors linked to ECMO-IC that may have remained undetected in previous multivariable logistic regression models [11]. Recent progress in computational power and data analysis capabilities has enabled ML implementations that exceed traditional methodologies in resolving complex clinical judgment scenarios [12], representing a vital advantage in the contemporary era of precision medicine. Two ML models [13, 14] have been developed to predict hemorrhage and thrombosis during ECMO but not ECMO-IC. Furthermore, considering that the sample sizes of the studies were limited [11], the models' generalizability and stability require further validation, while the overall methodological quality (feature selection, overfitting issues, cross-validation, model comparison and model interpretation) was inadequate [12]. Most critically, existing models are inappropriate for delivering early decision support to prevent ECMO-IC by identifying high-risk ECMO-IC cases, since all models were constructed using ECMO patients, incorporating post-ECMO variables.

This retrospective study sought to establish a sophisticated multi-criteria decision framework utilizing leave-one-out cross-validation (LOOCV) methodology with 12 classical ML algorithms for the early prediction of ECMO-IC risk, employing routine clinical data collected before ECMO support initiation within 24 h. We hypothesized that an ML model that integrates comprehensive electronic medical records of pre-ECMO variables can achieve clinically superior accuracy and predictive performance. This model may provide clinicians with insights regarding modifiable factors linked to ECMO-IC outcomes and help identify ECMO patients at an elevated risk for coagulopathy, thus facilitating timely and personalized interventions to prevent and manage ECMO-IC.

## Materials and Methods

This study was approved (Approval No. KYLS20220606) by the Ethics Review Committee of the Eighth Affiliated Hospital, Southern Medical University (The First People’s Hospital of Shunde, Foshan), conforming to the ethical principles outlined in the World Medical Association Declaration of Helsinki. Given the retrospective nature of this study and the utilization of deidentified patient data, the requirement for informed patient consent was exempted. The study was registered in the Chinese Clinical Trial Registry (chictr.org.cn), ChiCTR2200062824 (https://www.chictr.org.cn/showproj.html?proj=177223). This study per the Transparent Reporting of a Multivariable Prediction Model for Individual Prognosis or Diagnosis (TRIPOD) + artificial intelligence (AI) guidelines [15].

### Study design

**Fig. 1:**
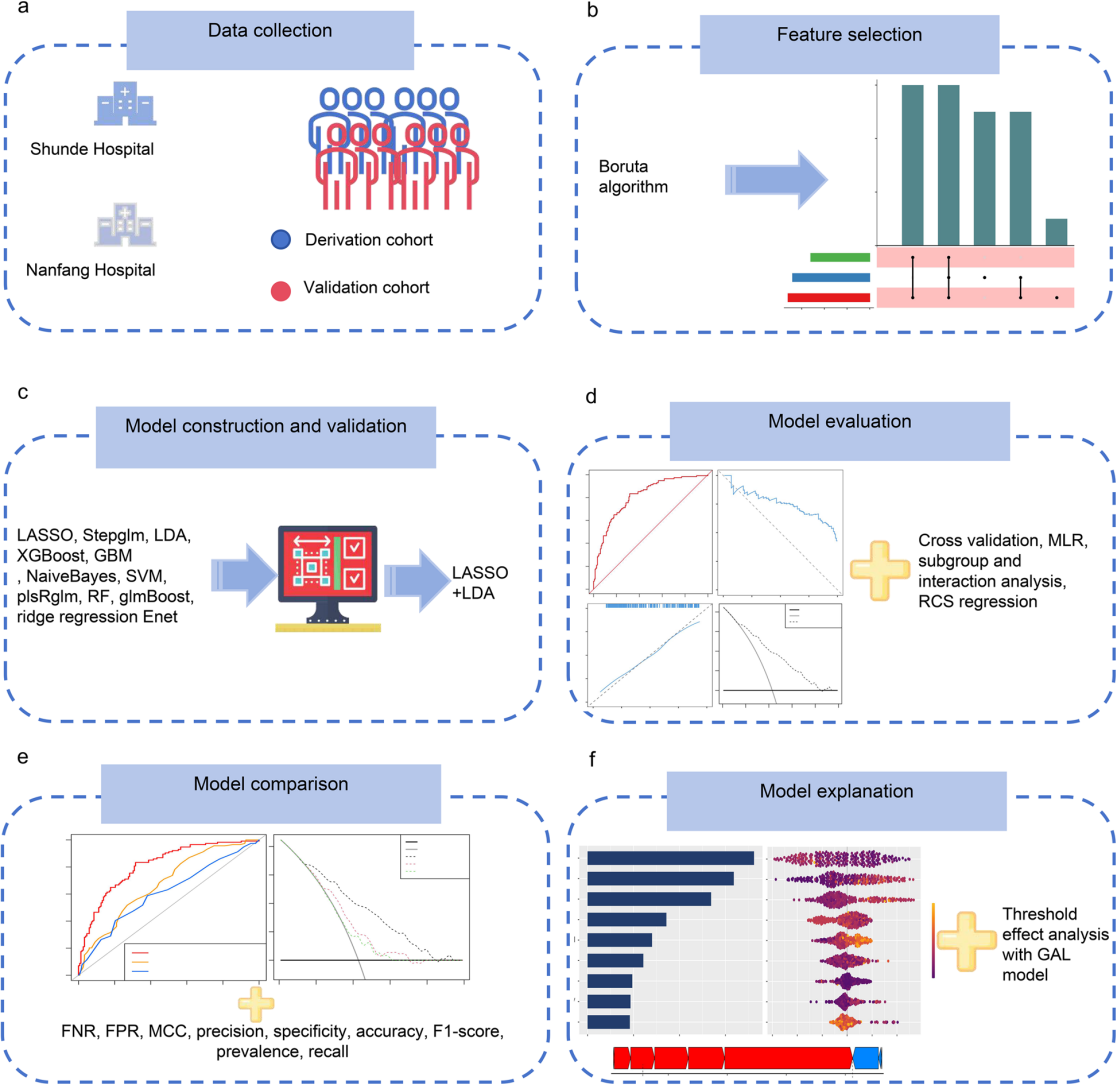
The illustrations for this study

This study follows a 6-phase methodology: data acquisition, variable selection, model development and internal validation, model assessment, model comparison, and model explanation. The initial phase involved extracting the clinical records of eligible ECMO patients from two hospitals. Subsequently, the Boruta algorithm identified relevant features, then proceeded with the implementation of an advanced LOOCV framework incorporating 12 ML approaches, producing 105 combinations to establish a consensus prediction model. Additionally, the assessment phase examined model effectiveness, discriminative ability, consistency, calibration accuracy and clinical applicability. Ultimately, the Shapley Additive Explanations (SHAP) algorithm provided local and global model interpretations, while restricted cubic spline (RCS) regression and threshold effect analysis with generalized additive linear model revealed nonlinear relationships between ECMO-IC and predictors, identifying critical threshold points. The overall workflow of this study is drawn in Fig. 1.

### Study population

This multicenter retrospective analysis encompassed patients undergoing ECMO at The Eighth Affiliated Hospital, Southern Medical University (January 2015 to December 2024) and Nanfang Hospital, Southern Medical University (January 2018 to December 2021). Indications for ECMO, including Veno-arterial (VA)-ECMO and veno-venous (VV)-ECMO, followed the ELSO (Extracorporeal Life Support Organization) guidelines [16]. VV-ECMO focuses on respiratory support, with cannulation prioritising minimised recirculation; VA-ECMO emphasises circulatory support, requiring cannulation to balance arterial perfusion and distal limb perfusion.

Inclusion criteria: 1) Patients aged ≥ 18 years; 2) Patients undergoing ECMO support ≥ 24 h; and 3) The missing data of the patients < 20%. The exclusion criteria were as follows: 1) Patients with coagulation disorders before ECMO; 2) Anticoagulation treatment before ECMO; 3) Pregnancy; 4) Existing malignant tumor; and 5) Death within 24 h of ECMO treatment. In cases where patients underwent multiple ECMO treatments, only the first occurrence was included in the analysis.

Following these criteria, 266 eligible ECMO patients were included in this study, of which 203 were from Shunde Hospital and 63 were from Nanfang Hospital. Based on the time of ECMO treatment, the subjects were sequentially distributed into a derivation cohort (January 2021 to December 2024; n = 125) and an internal validation cohort (January 2015 to December 2020; n = 141).

### Data collection and preprocessing

For this cohort, data were gathered regarding demographic characteristics, past medical history, indications for using ECMO, ECMO type, parameters of initial ECMO settings; vital signs, arterial blood gas, biochemical indicators, and disease severity within 24 h prior to ECMO initiation; treatments and medications administered during ECMO (albumin use, continuous renal replacement therapy (CRRT), vasoactive drugs (VAD)); clinical outcome (survival to discharge, mechanical ventilation (MV) time, ECMO treatment time, ICU/hospital length of stay (LOS)). The 3σ method was used to detect outliers in the static data that were treated as missing data. To address missing values, we utilized multiple imputation with chained equations (MICE), assuming that missing data occurred randomly [17]. All variables considered for inclusion in the multivariate model were incorporated into the input phase, and Rubin's rule was used to summarize the results from the 10 input datasets. These preprocessing steps are performed once for the entire dataset. The proportion of missing data for variables is provided in Supplementary Material 1.

### ECMO-related coagulopathy definition

Coagulopathy was diagnosed during the ECMO therapy period based on the latest International Society on Thrombosis and Haemostasis (ISTH) Scientific and Standardization Committee (SSC) [8, 18]. The criteria include: Platelet count: 1 point if ≤ 50–100 × 10^9^/L; 2 points if < 50 × 10^9^/L. prothrombin time/international normalized ratio (PT/INR): 1 point if ≥ 3–6 s; 2 points if ≥ 6 s. D2 dimer: 2 points if > 3 times the upper normal limit; 3 points if ≥ 7 times the upper normal limit; fibrinogen (FIB): 1 point if < 1 g/L. Patients were diagnosed with ECMO-related coagulopathy if the total score reached 5 or more.

### Statistical analysis

#### Description and comparison of clinical features

Normally distributed continuous data are presented as mean ± standard deviation (SD), with between-group analyses conducted via an independent samples t-test. Regarding non-normal distributions, median values along the interquartile range (IQR, p25–p75) were documented, while group assessments utilized the Mann–Whitney U test. Categorical variables are displayed as frequencies (percentages, %) and analyzed using the chi-square test or Fisher's exact test when applicable.

#### Feature selection

The selection of relevant variables serves a fundamental function in optimizing the performance of the diagnostic model. Therefore, the Boruta algorithm, which utilizes a random forest classifier to perform feature selection across multiple datasets with high-dimensional and multivariate characteristics, was implemented [19].

#### Model development and assessment

Using feature variables identified using the Boruta algorithm, a consensus diagnostic framework for ECMO-IC was developed and assessed. The methodology was as follows.

1. ML algorithms combinations: Initially, 12 standard ML algorithms were included Least Absolute Shrinkage and Selection Operator (LASSO), Stepglm, Linear Discriminant Analysis (LDA), extreme Gradient Boosting (XGBoost), gradient boosting machine (GBM), NaiveBayes, SVM, partial least squares regression for generalized linear models (plsRglm), random forest (RF), glmBoost, ridge regression, and elastic network (Enet). By combining 12 ML algorithms with parameter optimization, 105 different combinations were generated as prediction frameworks using the LOOCV structure.
2. Model developing: Among these, Stepglm, RF, LASSO, and glmBoost possess feature-selection capabilities. The resampling scheme employed in feature selection is fivefold cross-validation in derivation cohort. Feature selection was performed inside each fold. First select the feature variables, then train the model. For a combined model, the former method selects features while the latter performs modelling.
3. Model evaluation: Subsequently, 105 ML combinations were evaluated using the area under the curve (AUC) in the derivation cohort. AUC calculations were performed for each model obtained from the derivation cohort within the validation cohort.
4. Model determination: The definitive consensus diagnostic framework for ECMO-IC (ECMO-IC index) was determined using the average AUC of both cohorts, considering model fit and simplicity.

#### Model evaluation

To evaluate the degree of multicollinearity among variables within the ECMO-IC index, the variance inflation factor (VIF) was computed using multiple linear regression analysis. If the VIF value exceeded 5 or the tolerance was less than 0.2, multicollinearity was considered high. Additionally, Spearman correlation analysis was conducted to assess multicollinearity issues. When the correlation coefficient between two variables exceeds 0.5 in Spearman's correlation analysis, it is considered highly correlated, indicating the presence of multicollinearity.

The discriminative performance of the ECMO-IC index was evaluated using receiver operating characteristic (ROC) and precision recall (PR) analyses of the AUC values. Bootstrap with 1000 resamples was used for validation to calculate the corrected AUC. The model's predictive performance was assessed through multiple comprehensive metrics, including sensitivity, specificity, positive predictive value (PPV), negative predictive value (NPV), false negative rate (FNR), false positive rate (FPR), Matthews correlation coefficient (MCC), precision, accuracy, F1-score, prevalence, recall. To assess the ECMO-IC index calibration, a calibration plot was constructed to demonstrate the correspondence between predicted and observed average probabilities. To establish the clinical utility of the ECMO-IC index, decision curve analysis (DCA) was performed through net benefit calculations across different threshold probabilities by evaluating and comparing the ECMO-IC index with the baseline.

Finally, Bootstrap methodology and fivefold cross-validation techniques were employed to assess the reliability and stability capacity of the ECMO-IC index [20]. Cross-validation is an analytical method that validates model results by dividing a dataset into training and testing datasets, thus enabling evaluation and comparison of various model performances on limited datasets. Through cross-validation, researchers can prevent dependence on singular experiments and more effectively assess the model’s stability capacity.

#### Sensitivity analysis

To investigate whether performing missing data imputation on the entire dataset before splitting might lead to data leakage and over-optimistic results. Sensitivity analyses were performed to exclude participants with any missing data in derivation cohort (n = 107), validation cohort (n = 121), and entire cohort (n = 228).

#### Forward-time validation

To justify this choice that the derivation cohort (2021–2024) postdates the validation cohort (2015–2020). We performed a forward-time validation, namely derivation cohort (2015–2020) and validation cohort (2021–2024). ECMO-IC was developed and assessed using an innovative LOOCV framework.

#### Multivariable logistic regression, subgroup analysis and interaction effect

Multivariable logistic regression was applied to determine whether the ECMO-IC index was an independent risk indicator of coagulopathy after ECMO. To investigate potential differences within particular groups, subgroup analysis was executed by categorizing patients based on medical institutions, survival outcome, sequential organ failure assessment (SOFA) score, CRRT, sex, age, indications for using ECMO, ECMO type, ever experienced cardiopulmonary resuscitation(CPR), albumin use, VAD use (≥ 2), MV time, ECMO treatment time, diabetes mellitus (DM), hypertension (HBP), respiratory system diseases (RSD), cardiovascular system diseases (CSD), liver diseases (HD), renal diseases (RD), nervous system diseases (NSD), digestive system diseases (DSD). The interaction between these variables and ECMO-IC index employed for stratification in subgroup analysis was assessed through likelihood ratio test. To account for multiple testing and avoid false-positive results, Benjamini–Hochberg correction was applied in subgroup analyses, with the significance threshold set at an adjusted *P* value < 0.05.

#### Model comparison

Multiple ROC analysis was conducted to assess and compare the discriminative performance of the ECMO-IC index against acute physiology and chronic health evaluation II (APACHE II) and SOFA. DCA was employed to determine the net benefit of the ECMO-IC index versus APACHE II and SOFA. The ECMO-IC model’s predictive capabilities against APACHE II and SOFA underwent assessment through FNR, FPR, MCC, precision, specificity, accuracy, F1-score, prevalence, recall.

#### Model interpretability

To facilitate interpretation of the model, the SHAP method [21] was employed. SHAP values [22] were computed to assess the contribution of each clinical variable and to quantitatively describe the overall association between all 9 features and ECMO-IC in the pre-established model. Additionally, the SHAP methodology provides local and global explanations of the model. Local interpretation can display specific predictions of the risk of coagulation dysfunction in individual patients receiving ECMO by entering specific data. Global interpretation delivers consistent and precise attribution scores for each feature within the model, highlighting their relationship with ECMO-IC. The SHAP dependency graph shows the nonlinear interactive relationship between the predictor feature and ECMO-IC.

#### Logistic regression, RCS regression and threshold effect analysis:

To examine the relevance of parameters in the ECMO-IC model, both linear and nonlinear relationships between ECMO-IC and predictive factors were investigated. Linear correlations were evaluated through single-variable logistic regression analyses, with individual clinical parameters serving as predictors for ECMO-IC. Wald tests were used to test regression coefficients for linearity. Additionally, the potential nonlinear associations between ECMO-IC and predictive variables were evaluated using RCS regression with chi-square tests. Finally, threshold effect analysis with generalized additive linear model was performed to yield inflection point for predictive variables, thereby guiding clinicians in making clinical decisions.

Statistical analyses were executed using R software (R version 4.3.1), SPSS statistics 22.0, and DecisionLinnc1.0 software (Python). DecisionLinnc1.0 serves as an integrated platform that combines multiple programming environments and utilizes a visual interface for data processing and analysis [23]. A two-sided *P* < 0.05 was considered significant.

### Visualized online calculator

To provide a more flexible forecasting tool for ECMO-IC model, an online calculator with a visual interface was developed to facilitate the easy input of clinical variables and to generate clear and meaningful output indicating the ECMO-IC risk.

## Results

### Study population and patient characteristics

Coagulopathy after patients receiving ECMO was 88 (88/203 = 43.35%) in Shunde Hospital, 26 (26/63 = 41.27%) in Nanfang Hospital, 51 (51/125 = 40.80%) in derivation cohort and 61 (61/141 = 44.68%) in validation cohort. We compared several baseline characteristics and ECMO-IC incidence (40.80% *vs* 44.68%; *P* = 0.523) (Supplementary Material 2) between derivation cohort and validation cohort. No significant differences in those clinical characteristics were detected except for VAD (≥ 2) usage (*P* = 0.005).

Subsequently, we compared 75 routine clinical features between the coagulopathy and the non-coagulopathy groups in the derivation cohort, validation cohort, and entire cohort (Table 1). Compared to the non-coagulopathy group, it was observed that CRRT/albumin/VAD (≥ 2) usage and ICU mortality were markedly elevated in the coagulopathy group.

**Table 1:** Comparison of baseline characteristics and biochemical indicators between coagulopathy and non-coagulopathy groups in ECMO patients in derivation, validation and entire cohorts

Cohort		Derivation cohort			Validation cohort			Entire cohort			
Variable	Levels	Non-coagulopathy	Coagulopathy	*P*	Non-coagulopathy	Coagulopathy	*P*	Overall	Non-coagulopathy	Coagulopathy	*P*
		n = 74	n = 51		n = 78	n = 63		n = 266	n = 152	n = 114	
Age (y), mean (SD)		46.84 (17.15)	50.02 (17.61)	0.318	51.05 (14.50)	47.65 (19.24)	0.248	48.88 (17.04)	49 (15.93)	48.71 (18.49)	0.894
Sex, n (%)				0.290			0.317				0.969
	Male	47 (63.51)	37 (72.55)		58 (74.36)	42 (66.67)		184 (69.17)	105 (69.08)	79 (69.30)	
	Female	27 (36.49)	14 (27.45)		20 (25.64)	21 (33.33)		82 (30.83)	47 (30.92)	35 (30.70)	
Indications, n (%)				0.078			0.016				0.004
	AM	22 (29.73)	7 (13.73)		10 (12.82)	4 (6.35)		43 (16.17)	32 (21.05)	11 (9.65)	
	ARDS	5 (6.76)	6 (11.76)		12 (15.38)	8 (12.70)		31 (11.65)	17 (11.18)	14 (12.28)	
	CPR	1 (1.35)	2 (3.92)		5 (6.41)	17 (26.98)		25 (9.40)	6 (3.95)	19 (16.67)	
	DM	0 (0)	0 (0)		2 (2.56)	1 (1.59)		3 (1.13)	2 (1.32)	1 (0.88)	
	ICM	0 (0)	3 (5.88)		1 (1.28)	4 (6.35)		8 (3.01)	1 (0.66)	7 (6.14)	
	MI	25 (33.78)	17 (33.33)		33 (42.31)	15 (23.81)		90 (33.83)	58 (38.16)	32 (28.07)	
	PTE	5 (6.76)	1 (1.96)		0 (0)	3 (4.76)		9 (3.38)	5 (3.29)	4 (3.51)	
	SP	8 (10.81)	5 (9.80)		3 (3.85)	2 (3.17)		18 (6.77)	11 (7.24)	7 (6.14)	
	VHD	1 (1.35)	2 (3.92)		0 (0)	0 (0)		3 (1.13)	1 (0.66)	2 (1.75)	
	MTT	0 (0)	3 (5.88)		2 (2.56)	1 (1.59)		6 (2.26)	2 (1.32)	4 (3.51)	
	SA	1 (1.35)	0 (0)		1 (1.28)	2 (3.17)		4 (1.50)	2 (1.32)	2 (1.75)	
	Other	6 (8.11)	5 (9.80)		9 (11.54)	6 (9.52)		26 (9.77)	15 (9.87)	11 (9.65)	
ECMO, n (%)				0.477			0.202				0.672
	VA	57 (77.03)	38 (74.51)		56 (71.79)	52 (82.54)		203 (76.32)	113 (74.34)	90 (78.95)	
	VV	17 (22.97)	12 (23.53)		20 (25.64)	11 (17.46)		60 (22.56)	37 (24.34)	23 (20.18)	
	VAV	0 (0)	1 (1.96)		2 (2.56)	0 (0)		3 (1.13)	2 (1.32)	1 (0.88)	
DM, n (%)				0.785			0.286				0.333
	No	58 (78.38)	41 (80.39)		60 (76.92)	53 (84.13)		212 (79.70)	118 (77.63)	94 (82.46)	
	Yes	16 (21.62)	10 (19.61)		18 (23.08)	10 (15.87)		54 (20.30)	34 (22.37)	20 (17.54)	
HBP, n (%)				0.720			0.994				0.785
	No	50 (67.57)	36 (70.59)		57 (73.08)	46 (73.02)		189 (71.05)	107 (70.39)	82 (71.93)	
	Yes	24 (32.43)	15 (29.41)		21 (26.92)	17 (26.98)		77 (28.95)	45 (29.61)	32 (28.07)	
RSD, n (%)				0.086			0.828				0.233
	No	74 (100)	49 (96.08)		76 (97.44)	61 (96.83)		260 (97.74)	150 (98.68)	110 (96.49)	
	Yes	0 (0)	2 (3.92)		2 (2.56)	2 (3.17)		6 (2.26)	2 (1.32)	4 (3.51)	
CSD, n (%)				0.844			0.911				0.999
	No	69 (93.24)	48 (94.12)		71 (91.03)	57 (90.48)		245 (92.11)	140 (92.11)	105 (92.11)	
	Yes	5 (6.76)	3 (5.88)		7 (8.97)	6 (9.52)		21 (7.89)	12 (7.89)	9 (7.89)	
HD, n (%)				0.790			0.268				0.254
	No	73 (98.65)	50 (98.04)		76 (97.44)	59 (93.65)		258 (96.99)	149 (98.03)	109 (95.61)	
	Yes	1 (1.35)	1 (1.96)		2 (2.56)	4 (6.35)		8 (3.01)	3 (1.97)	5 (4.39)	
RD, n (%)				0.790			0.757				0.743
	No	72 (97.30)	50 (98.04)		72 (92.31)	59 (93.65)		253 (95.11)	144 (94.74)	109 (95.61)	
	Yes	2 (2.70)	1 (1.96)		6 (7.69)	4 (6.35)		13 (4.89)	8 (5.26)	5 (4.39)	
NSD, n (%)				0.091			0.052				0.642
	No	70 (94.59)	51 (100)		77 (98.72)	58 (92.06)		256 (96.24)	147 (96.71)	109 (95.61)	
	Yes	4 (5.41)	0 (0)		1 (1.28)	5 (7.94)		10 (3.76)	5 (3.29)	5 (4.39)	
DSD, n (%)							0.828				0.771
	No	74 (100)	51 (100)		76 (97.44)	61 (96.83)		262 (98.50)	150 (98.68)	112 (98.25)	
	Yes	0 (0)	0 (0)		2 (2.56)	2 (3.17)		4 (1.50)	2 (1.32)	2 (1.75)	
Other, n (%)				0.537			0.312				0.254
	No	69 (93.24)	46 (90.20)		74 (94.87)	57 (90.48)		246 (92.48)	143 (94.08)	103 (90.35)	
	Yes	5 (6.76)	5 (9.80)		4 (5.13)	6 (9.52)		20 (7.52)	9 (5.92)	11 (9.65)	
SOFA, mean (SD)		8.95 (4.27)	9.35 (4.55)	0.615	9.01 (3.80)	11.62 (3.97)	< 0.001	9.68 (4.24)	8.98 (4.02)	10.61 (4.37)	0.002
APACHEII, mean (SD)		21.11 (9.41)	27.51 (9.35)	< 0.001	22.82 (10.30)	28.81 (9.82)	< 0.001	24.66 (10.22)	21.99 (9.88)	28.23 (9.59)	< 0.001
CPR (h), median (p25—p75)		20 (0—51)	20 (0—65)	0.103	20 (0—45)	20 (0—90)	0.052	20 (0—59)	20 (0—49.50)	20 (0—70)	0.011
First speed (r/min ), mean (SD)		3,047.45 (531.39)	2,943.33 (519.86)	0.278	3,032.17 (507.68)	3,077.44 (685.83)	0.663	3,030.11 (561.94)	3,039.61 (517.69)	3,017.45 (618.18)	0.757
FF (L/min ), mean (SD)		2.97 (0.77)	2.90 (0.88)	0.662	2.84 (0.84)	2.85 (0.82)	0.939	2.89 (0.82)	2.90 (0.80)	2.88 (0.84)	0.778
O_2_, median (p25—p75)		100 (80—100)	100 (80—100)	0.828	100 (80—100)	100 (100—100)	0.333	100 (80—100)	100 (80—100)	100 (100—100)	0.577
AF (L/min ), mean (SD)		2.99 (1)	3.16 (1.25)	0.419	3.27 (1.48)	3.93 (1.86)	0.023	3.32 (1.46)	3.13 (1.27)	3.58 (1.65)	0.016
HR(beat), mean (SD)		95.09 (28.74)	101.82 (32.73)	0.238	109.17 (26.82)	107.19 (29.30)	0.680	103.38 (29.54)	102.32 (28.57)	104.79 (30.86)	0.505
RR(times), mean (SD)		17.09 (5.79)	17.78 (7.04)	0.565	17.86 (5.87)	18.10 (6.40)	0.822	17.69 (6.19)	17.49 (5.82)	17.96 (6.67)	0.550
SBP(mmHg), mean (SD)		99.74 (23.11)	86.67 (27.64)	0.007	97.44 (27.13)	87.97 (33.97)	0.075	93.77 (28.40)	98.56 (25.20)	87.39 (31.17)	0.002
DBP(mmHg), mean (SD)		68.14 (15.05)	62.10 (17.24)	0.046	66.04 (16.29)	61.22 (17.74)	0.099	64.73 (16.65)	67.06 (15.68)	61.61 (17.45)	0.009
MAP(mmHg), mean (SD)		78.48 (15.01)	70.87 (19.26)	0.020	76.47 (17.70)	69.68 (20.66)	0.041	74.35 (18.35)	77.45 (16.42)	70.21 (19.97)	0.002
SI, mean (SD)		1.03 (0.54)	1.28 (0.61)	0.019	1.21 (0.47)	1.38 (0.63)	0.081	1.21 (0.57)	1.12 (0.51)	1.33 (0.62)	0.003
PH, mean (SD)		7.34 (0.14)	7.27 (0.22)	0.074	7.30 (0.17)	7.23 (0.24)	0.058	7.29 (0.20)	7.32 (0.16)	7.25 (0.23)	0.008
Lac(mmol/L), mean (SD)		5.57 (5.45)	9.32 (6.35)	< 0.001	7.32 (7.11)	12.17 (6.64)	< 0.001	8.36 (6.85)	6.47 (6.40)	10.90 (6.64)	< 0.001
HCO_3_-(mmol/L), mean (SD)		22.96 (6.05)	22.99 (8.03)	0.983	22.14 (8.25)	18.25 (7.81)	0.005	21.61 (7.74)	22.54 (7.25)	20.37 (8.22)	0.026
PaCO_2_(mmHg), median (p25—p75)		40.50 (32—49)	45 (34—60)	0.028	38.20 (32.70—56)	41 (32.90—52.40)	0.901	41 (32.80—54.90)	39.95 (32.60—53)	42.15 (33—56.80)	0.104
PaO_2_(mmHg), median (p25—p75)		79.50 (52—182)	77 (57—164)	0.666	76.90 (52—171)	78 (52—188)	0.299	77.25 (52—179)	76.90 (52—180.50)	77.50 (57—164)	0.311
OI(mmHg), median (p25—p75)		172.50 (60.80—368)	132.50 (70.90—328)	0.950	94.20 (59—253)	99 (63—164)	0.907	111 (62.30—311)	115.50 (59.50—312)	107.50 (65—297)	0.916
WBC(× 10^9^ /L), median (p25—p75)		13.99 (10.91—21.12)	16.12 (8.66—23.70)	0.376	13 (9.05—17.40)	12.83 (9.17—17.07)	0.293	13.67 (9.79—19.35)	13.19 (10.03—18.80)	13.95 (9.17—19.97)	0.956
NEU(× 10^9^ /L), median (p25—p75)		10.74 (7.50—16.24)	10.60 (5.95—19.05)	0.761	10.23 (7.66—14.69)	9.34 (5.85—12.65)	0.034	10.28 (7.25—15.67)	10.45 (7.55—15.40)	9.80 (5.95—15.72)	0.239
PLT(× 10^9^ /L), median (p25—p75)		208 (147—266)	121 (69—201)	0.015	189 (124—254)	111 (44—169)	< 0.001	160 (100—228)	195 (135—260)	114 (55—187)	< 0.001
RBC(× 10^9^ /L), mean (SD)		3.98 (1.24)	3.65 (1.15)	0.131	3.14 (0.76)	2.91 (0.70)	0.058	3.42 (1.07)	3.55 (1.10)	3.24 (0.99)	0.017
LY(× 10^9^ /L), median (p25—p75)		1.35 (0.91—2.21)	1.66 (1.21—3.23)	0.046	1.63 (1.05—2.72)	1.81 (0.97—3.16)	0.786	1.61 (0.99—2.94)	1.58 (0.96—2.52)	1.80 (1.06—3.22)	0.329
Hb(g/L), mean (SD)		112.54 (35.62)	114.75 (29.96)	0.710	97.06 (24.74)	84.37 (19.48)	< 0.001	101.75 (30.48)	104.60 (31.40)	97.96 (28.90)	0.075
HCT(%), mean (SD)		0.36 (0.10)	0.35 (0.09)	0.865	0.30 (0.07)	0.26 (0.06)	< 0.001	0.32 (0.09)	0.33 (0.09)	0.30 (0.09)	0.014
RDWSD(fL), mean (SD)		45.35 (8.26)	47.25 (9.17)	0.240	52.16 (10.06)	56.12 (10.52)	0.025	50.26 (10.38)	48.85 (9.81)	52.15 (10.85)	0.011
RDWCV(%), mean (SD)		14.71 (3.24)	15.05 (4.92)	0.661	16.04 (3.27)	18.18 (3.65)	< 0.001	15.99 (3.93)	15.39 (3.31)	16.78 (4.52)	0.006
PCT(%), median (p25—p75)		0.21 (0.15—0.27)	0.18 (0.11—0.26)	0.551	0.20 (0.10—0.28)	0.06 (0.03—0.13)	< 0.001	0.17 (0.08—0.25)	0.20 (0.13—0.27)	0.11 (0.04—0.22)	< 0.001
PDW(%), mean (sd)		10.62 (1.18)	10.69 (1.27)	0.743	11.28 (1.26)	11.71 (1.74)	0.103	11.08 (1.43)	10.96 (1.26)	11.25 (1.62)	0.106
NLR, median (p25—p75)		7.55 (4.49—16.15)	5.39 (2.58—10.72)	0.180	6.75 (3.52—12.15)	5.05 (2.28—8.89)	0.526	6.23 (3.02—11.58)	7.09 (4.01—13.83)	5.09 (2.33—9.25)	0.128
PLR, median (p25—p75)		136.27 (80.84—238.54)	74.81 (20.78—145.19)	0.008	116.97 (47.80—182.46)	63.93 (22.37—114.77)	0.029	97.66 (43.78—179.17)	124.03 (68.39—217.46)	69.34 (21.62—134.25)	< 0.001
SII, median (p25—p75)		1,435.70 (834.74—3,475.84)	731 (252.38—1,440.71)	0.010	1,267.81 (386.73—2,640.30)	591.51 (129.44—1,353.26)	0.026	1,054.87 (307.48—2,303.69)	1,373.70 (624.22—3,008.98)	602.79 (194.11—1,404.62)	< 0.001
Ca(mmol/L), mean (SD)		1.06 (0.18)	1 (0.19)	0.099	0.99 (0.14)	0.96 (0.19)	0.400	1 (0.18)	1.02 (0.16)	0.98 (0.19)	0.063
K(mmol/L), mean (SD)		4.05 (0.73)	4.41 (1.19)	0.057	3.92 (0.67)	4.44 (1.37)	0.007	4.17 (1.02)	3.98 (0.70)	4.43 (1.29)	0.001
Na(mmol/L), mean (SD)		140.93 (6.27)	143.09 (7.85)	0.106	141.82 (6.67)	145.02 (9.32)	0.024	142.57 (7.62)	141.39 (6.48)	144.15 (8.71)	0.005
ProCT(ng/mL), median (p25—p75)		3.38 (0.40—21.30)	2.83 (0.67—13.12)	0.512	2.77 (0.50—12.76)	5.31 (1.02—30.13)	0.234	3.51 (0.55—19.62)	2.91 (0.44—19.10)	4.37 (0.74—19.78)	0.813
CRP(ng/L), median (p25—p75)		39.40 (12—104.40)	12.50 (5—53.82)	0.131	26.50 (8—95.26)	36.80 (5—80.43)	0.147	27.35 (7.20—87.16)	34.30 (9.24—101.66)	18.09 (5—72.70)	0.040
ALT(U/L) , median (p25—p75)		79.50 (33—204)	220 (54—677)	0.021	74 (33—143)	141 (43—950)	< 0.001	101 (38—331)	75 (33—193.50)	167.50 (52—900)	< 0.001
AST(U/L), median (p25—p75)		227.50 (59—799)	544 (96—1,724)	0.032	158 (49—572)	479 (83—2,510)	0.008	241 (61—930)	178.50 (54—692.50)	500.50 (91—1,820)	< 0.001
LDH(U/L), median (p25—p75)		701.50 (419—1,719)	901 (371—2,001)	0.527	556 (278—1,174)	965 (451—3,290)	0.001	698 (332—1,906)	619 (313.50—1,452.50)	916.50 (388—2,801)	0.002
TBIL(μmol/L), median (p25—p75)		13.29 (9.94—27.39)	17.81 (9.76—30.70)	0.046	16.21 (10.36—25.20)	18.40 (10.56—35.50)	0.342	15.54 (10.20—28.82)	14.98 (10.17—26.44)	18.27 (10.56—33.90)	0.024
DBIL(μmol/L), median (p25—p75)		5.73 (3.34—12.59)	9.05 (4.75—17.10)	0.070	7.81 (4—15.51)	7.50 (5.40—18.90)	0.051	7.40 (4.04—15.52)	6.80 (3.76—13.72)	7.85 (4.83—17.93)	0.019
IBIL(μmol/L), median (p25—p75)		6.72 (4.96—11.21)	7.50 (5.42—14.70)	0.156	7.68 (5—11.40)	9.60 (5.80—15.60)	0.020	7.57 (5.03—13.08)	7.05 (4.98—11.31)	8.45 (5.80—15.50)	0.035
TP(g/L) , mean (SD)		54.76 (10.89)	49.54 (11.25)	0.011	53.92 (9.12)	48.73 (10.78)	0.003	52.08 (10.72)	54.33 (10)	49.09 (10.95)	< 0.001
ALB(g/L), mean (SD)		32.37 (6.96)	30.56 (8.27)	0.202	33.50 (6.27)	32.88 (9.15)	0.646	32.47 (7.64)	32.95 (6.62)	31.84 (8.81)	0.260
BNP(pg/mL), median (p25—p75)		2,793.89 (682.70—9,160)	4,060 (1,930—15,300)	0.780	936.35 (137.70—6,397)	1,822 (320.10—7,678)	0.214	2,150.50 (359—8,776)	2,015 (281.50—7,735)	2,531 (648.30—11,148)	0.927
CK(U/L), median (p25—p75)		764 (120—3,955)	958 (153—6,614)	0.957	1,764.50 (270—6,080)	1,468 (155—4,865.30)	0.596	1,259 (218—5,120)	1,403 (231—4,707)	1,195.50 (155—6,200)	0.748
CKMB(U/L), median (p25—p75)		65.70 (23.70—290.50)	59 (21—331)	0.758	100 (22—336)	175 (36—500)	0.210	84.35 (24.70—336)	77 (23—318.80)	89.40 (27—430)	0.843
Cr(μmol/L), median (p25—p75)		114.40 (76—158.10)	132 (96.80—216.70)	0.022	119.85 (80—159)	139.20 (103—206.49)	0.231	124.05 (88—184)	115.30 (77.75—159)	139.10 (98.70—207)	0.016
PTT(s), median (p25—p75)		13.40 (11.80—17.80)	16 (13.40—31.10)	0.008	15.60 (13.10—19.20)	19.70 (15.80—24.60)	0.061	15.85 (13.10—21)	14.55 (12.40—18.75)	18.25 (14.70—26.40)	< 0.001
APTT(s), median (p25—p75)		34.05 (26.50—55.30)	38.10 (26.50—77.10)	0.490	48.70 (34.70—80)	66.30 (45.20—132.40)	0.285	46.40 (30.90—80)	43.40 (29.40—68.80)	50.40 (32.70—104.50)	0.167
TT(s), median (p25—p75)		19.65 (16.50—42.90)	25 (17.80—150)	0.113	20.55 (18.10—62.30)	25.90 (19.40—41.60)	0.774	22.10 (17.50—60)	20.50 (17.15—51.66)	25.80 (18.60—79.40)	0.315
FIB(g/L), median (p25—p75)		3.01 (2.03—4.40)	2.03 (1.43—4.49)	0.760	2.87 (1.95—3.67)	1.58 (1.15—2.71)	< 0.001	2.61 (1.49—3.79)	2.95 (2.03—3.95)	1.78 (1.17—3.11)	0.366
CRRT, n (%)				< 0.001			< 0.001				< 0.001
	No	42 (56.76)	11 (21.57)		42 (53.85)	9 (14.29)		104 (39.10)	84 (55.26)	20 (17.54)	
	Yes	32 (43.24)	40 (78.43)		36 (46.15)	54 (85.71)		162 (60.90)	68 (44.74)	94 (82.46)	
Albumin use, n (%)				0.622			0.029				0.174
	No	8 (10.81)	7 (13.73)		20 (25.64)	7 (11.11)		42 (15.79)	28 (18.42)	14 (12.28)	
	Yes	66 (89.19)	44 (86.27)		58 (74.36)	56 (88.89)		224 (84.21)	124 (81.58)	100 (87.72)	
VAD use(≥ 2), n (%)				0.861			0.036				0.143
	No	38 (51.35)	27 (52.94)		33 (42.31)	16 (25.40)		114 (42.86)	71 (46.71)	43 (37.72)	
	Yes	36 (48.65)	24 (47.06)		45 (57.69)	47 (74.60)		152 (57.14)	81 (53.29)	71 (62.28)	
Hospital LOS(d), median (p25—p75)		24.25 (11.77—41.64)	9.84 (3.08—28.58)	0.471	19.44 (10.05—30.27)	11.74 (4.90—21.03)	0.030	16.46 (8.05—29.86)	19.97 (10.90—32.13)	11.16 (3.84—21.88)	0.085
ICU LOS(d), median (p25—p75)		14.67 (7.73—27.98)	7.96 (2.36—17.39)	0.302	8.71 (5.92—15.88)	9.53 (3.77—15.93)	0.880	9.85 (4.99—18.76)	10.94 (6.46—22.09)	8.91 (3.46—16.01)	0.279
MVT(d), median (p25—p75)		8.65 (3.40—18.69)	7.78 (2.50—15.40)	0.940	6.47 (3.78—12.90)	8.97 (3.77—16.05)	0.573	7.80 (3.46—15.91)	7.42 (3.59—16.74)	8.53 (3.46—15.40)	0.817
ECMOT(d), median (p25—p75)		6.68 (3.29—12)	4.18 (1.92—8.14)	0.012	4 (2.48—6)	4.78 (2.93—9.12)	0.177	4.69 (2.73—8.97)	4.94 (2.96—9.22)	4.37 (2.25—8.77)	0.308
Outcome, n (%)				< 0.001			< 0.001				< 0.001
	Survival	43 (58.11)	9 (17.65)		44 (56.41)	11 (17.46)		107 (40.23)	87 (57.24)	20 (17.54)	
	Death	31 (41.89)	42 (82.35)		34 (43.59)	52 (82.54)		159 (59.77)	65 (42.76)	94 (82.46)	

*Abbreviations:* *ECMO* extracorporeal membrane oxygenation, *CRRT* continuous renal replacement therapy, *VA* venoarterial, *VV* venovenous, *VAV* venoarterial-venous, *DM* diabetes mellitus; HBP: hypertension, *RSD* respiratory system diseases, *CSD* cardiovascular system diseases, *HD* liver diseases, *RD* renal diseases, *NSD* nervous system diseases, *DSD* digestive system diseases, *AM* acute myocarditis, *ARDS* acute respiratory distress syndrome, *CPR* cardiopulmonary resuscitation, *DM* dermatomyositis, *ICM* cardiomyopathy, *MI* myocardial infarction, *PTE* pulmonary embolism, *SP* severe pneumonia, *VHD* heart valve disease, *MTT* multiple trauma, *SA* surgical assistance, *HR* heart rate, *SBP* systolic blood pressure, *DBP* diastolic blood pressure; SI: shock index; RR: respiratory rate, *SOFA* sequential organ failure assessment, *APACHEII* acute physiology and chronic health evaluation II, *Lac* lactate, *FF* first flow, *AF* air flow, *MAP* mean arterial pressure, *OI* oxygenation index, *WBC* white blood cell, *NEU* neutrophils, *PLT* platelet, *Hb* Hemoglobin, *Cr* creatinine, *ALT* alanine aminotransferase, *AST* aspartate aminotransferase, *LDH* lactate dehydrogenase, *TBIL* total bilirubin, *DBIL* direct bilirubin, *IBIL*, indirect bilirubin, *TP* total protein, *ALB* albumin, *PTT* prothrombin time, *APTT* activation partial thromboplastin time, *TT* thrombin time, *FIB* fibrinogen, *ProCT* procalcitonin, *BNP* brain natriuretic peptide, *CRP* C-reactive protein, *CK* creatine kinase, *CK-MB* creatine kinase isoenzymes, *SII* systemic immune-inflammation index, *NLR* neutrophil to lymphocyte ratio, *PLR* platelet to lymphocyte ratio, *HCT* hematocrit, *PCT* plateletocrit, *RBC* red blood cell, *RDW* red blood cell volume distribution width, *PDW* platelet distribution width, *LY* lymphocyte, *ICU* intensive care unite, *LOS* length of stay, *MVT* mechanical ventilation time, *ECMOT* extracorporeal membrane oxygenation time, *VAD* vasoactive drugs, *SD* standard deviation, *p25—p75* interquartile range percentage

Furthermore,the coagulopathy group was more likely to have higher SOFA, APACHE II, systolic blood pressure (SBP), diastolic blood pressure (DBP), shock index (SI), mean arterial pressure (MAP), lactate, K, Na, red blood cell volume distribution width (RDW), alanine aminotransferase (ALT), aspartate aminotransferase (AST), lactate dehydrogenase (LDH), total bilirubin (TBIL), direct bilirubin (DBIL), and indirect bilirubin (IBIL); longer CRP time and prothrombin time (PTT); and lower PH, HCO_3_-, platelet (PLT), hemoglobin (Hb), systemic immune-inflammation index (SII), red blood cell (RBC), platelet to lymphocyte ratio (PLR), hematocrit (HCT), plateletocrit (PCT), and total protein (TP). These observations indicated a significant correlation between the clinical parameters of pre-ECMO and the development of coagulopathy during ECMO.

### Variable selection

**Fig. 2:**
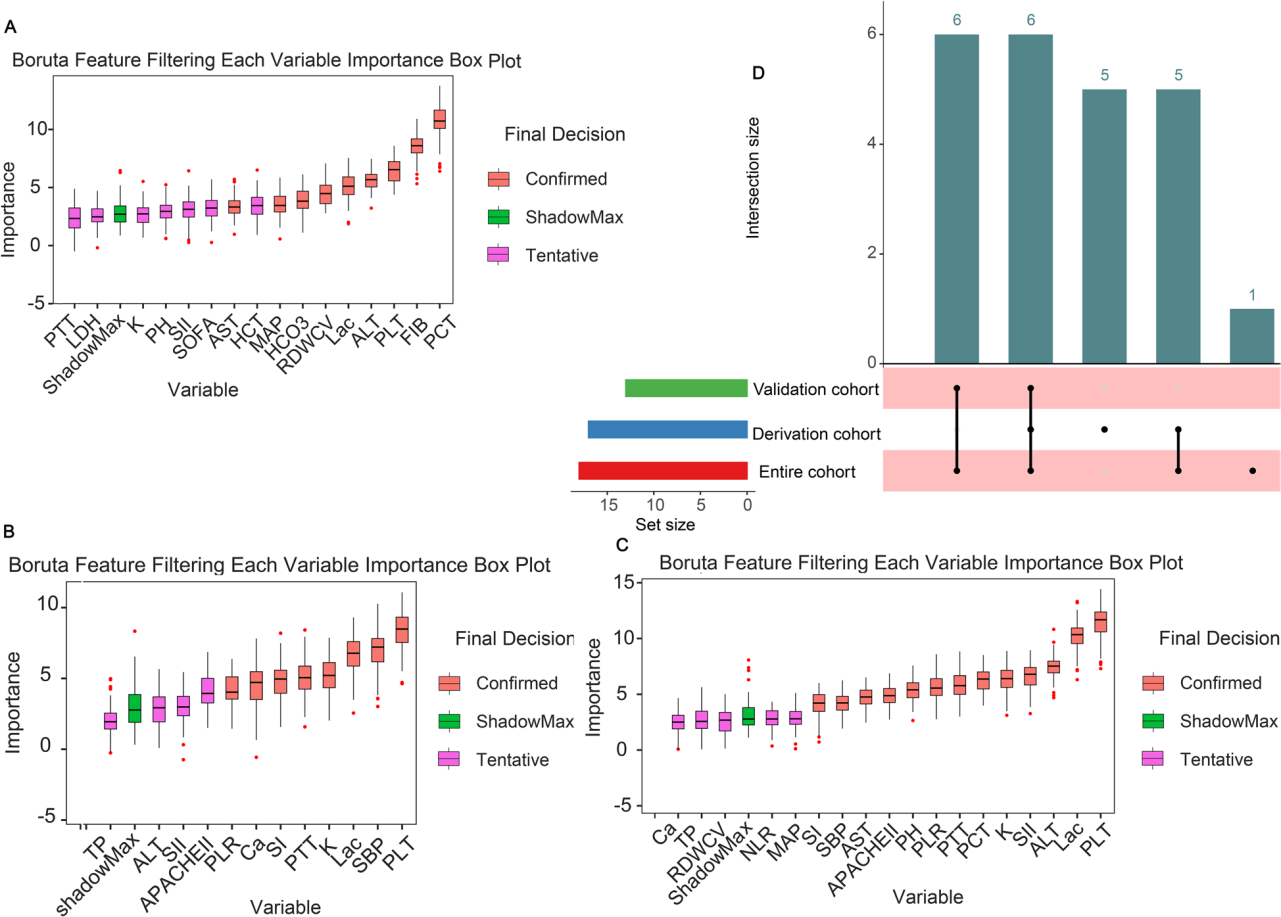
Important characteristic variables identified by the Boruta algorithm. The horizontal axis shows the names of variables, and the vertical axis shows the Z-score of variables. The boxplot shows the Z-score of variables during the model calculation process. **A** Derivation cohort. **B** Validation cohort. **C** Entire cohort. **D** UpSet diagram displays the shared feature variables in derivation, validation and entire cohorts

To address confounding elements and identify reliable variables, the Boruta algorithm enables a thorough assessment of clinical parameters. Through variable selection using the Boruta algorithm, 12 parameters emerged in derivation cohort (Fig. 2A), 16 parameters appeared in validation cohort (Fig. 2B), and 18 parameters emerged in entire cohort (Fig. 2C). Complete variable selection data are presented in Supplementary Material 3. Subsequently, the intersection of three results yielded 17 indicators (PLT, lactate, ALT, SII, K, PCT, PTT, TP, AST, PH, SBP, SI, RDWCV, APACHE II, Ca, MAP, PLR) shared by ≥ two or more results (Fig. 2D).

### Optimised model construction and validation

**Fig. 3:**
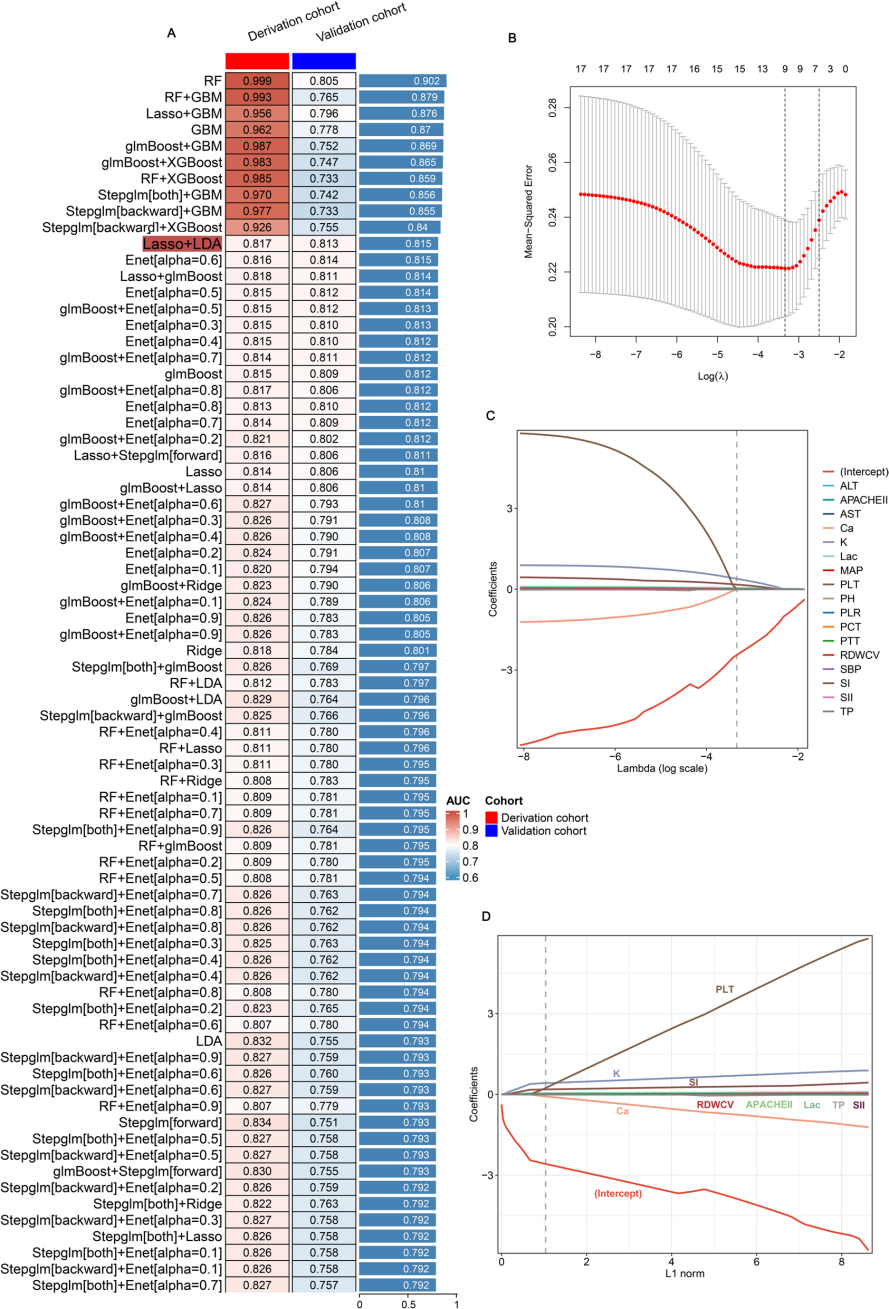
Establishment and validations of a consensus diagnostic model for ECMO-IC via 12 the machine learning (ML)-based integrative procedure. **A** A ML algorithm combinations of prediction models using the leave-one-out cross-validation (LOOCV) framework and further calculated the area under curve (AUC) of each model in derivation and validation cohorts. **B** Lasso was used to identify candidate features with fivefold cross-validation. The Y-axis shows mean-square error and the X-axis is Log (*λ*), dotted vertical lines represent minimum and 1 standard error values of *λ*. The features selected at minimum standard error values of *λ* were finally used for ECMO-IC model. **C**, **D** Lasso coefficient profiles of the 9 predictors. A vertical line is drawn at the optimal value by1—s.e. criteria and results in 9 non-zero coefficients (PLT, lactate, SII, K, TP, SI, RDWCV, APACHE II, Ca). PLT: platelet; SII: systemic immune-inflammation index; RDW: red blood cell volume distribution width; SI: shock index; TP: total protein; APACHE II: acute physiology and chronic health evaluation II

To develop an accurate diagnostic framework for predicting coagulopathy in patients under ECMO, 17 feature parameters were incorporated into the analytical system (LOOCV framework). The study established prediction frameworks utilizing 12 ML algorithms and 105 algorithmic combinations, applying fivefold cross-validation within the derivation group. The AUC values were calculated for individual algorithms and average AUC across both derivation and validation groups. The analysis revealed that "RF", "RF + GBM", “Lasso + GBM”, “GBM”, “glmBoost + GBM”, “glmBoost + XGBoost”, “RF + XGBoost”, “Stepglm[both] + GBM”,

“Stepglm[backward] + GBM” and “Stepglm[backward] + XGBoost” models exhibited substantial AUC values in the derivation group but showed decreased performance in the validation group, indicating overfitting. Although the performance of “Enet[alpha = 0.6]” is acceptable in both the derivation and validation cohorts, the number of feature variables is relatively high. After evaluating all aspects, the preferred model represented a combination of Lasso and LDA, exhibiting superior average AUC (0.815), reduced variability, and greater stability across both cohorts, as illustrated in Fig. 3A and Figure S1. Using the Lasso algorithm, the optimal λ (λ = 9; PLT, lactate, SII, K, TP, SI, RDWCV, APACHE II, Ca) was achieved when the mean − squared error attained its minimum value (Fig. 3B-D). The optimised diagnostic model (Lasso + LDA) was designated as the ECMO-IC index. Detailed information regarding feature selection, predictive classification, and individual risk scores is provided in Supplementary material 4.

### Model evaluation

First, VIF values below 2 and tolerance above 0.5 in derivation and validation cohorts indicated minimal multicollinearity among the variables in the ECMO-IC model (Supplementary material 5). The correlation coefficients between the predictor variables are all less than 0.5, further indicating that no substantial multi-collinearity issues (Supplementary material 5).

**Fig. 4:**
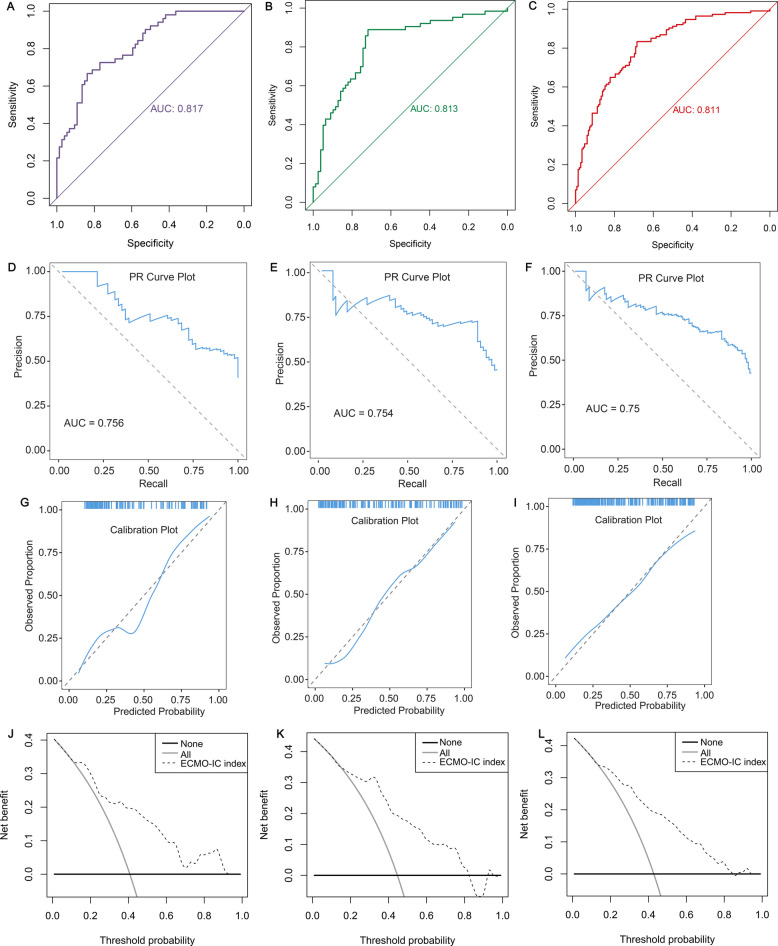
Evaluation of diagnostic value, fitting ability, clinical usefulness and nonlinear relationship of ECMO-IC index. **A**-**C** Receiver operating characteristic (ROC) curves for ECMO-IC index in derivation (**A**), validation (**B**) and entire (**C**) cohorts. **D**-**F** precision recall (PR) curves for ECMO-IC index in derivation (**D**), validation (**E**) and entire (**F**) cohorts. (G-I) Calibration curves for ECMO-IC index in derivation (**G**), validation (**H**) and entire (**I**) cohorts. X-axis is predicted probability of ECMO-IC. Y-axis is observed probability of ECMO-IC. (J-L) Decision curve analysis was applied to evaluate the clinical usefulness of ECMO-IC index in derivation (**J**), validation (**K**) and entire (**L**) cohorts. The Y-axis represents the net benefit. The black line represents the hypothesis that no patients treatment. The X-axis represents the threshold probability. The threshold probability is where the expected benefit of treatment is equal to the expected benefit of avoiding treatment

Second, ROC and PR curves revealed that the ECMO-IC index exhibited superior predictive performance, attaining an AUC of 0.817 (95%CI: 0.744–0.890) (Fig. 4A) and 0.756 (Fig. 4D) in derivation cohort, 0.813 (95%CI: 0.740–0.886) (Fig. 4B) and 0.754 (Fig. 4E) in validation cohort, 0.811 (95%CI: 0.760–0.863) (Fig. 4C) and 0.750 (Fig. 4F) in entire cohort, respectively. The bootstrap-adjusted AUC was 0.815 (95%CI: 0.742–0.89) in derivation cohort, 0.812 (95%CI: 0.744–0.890) in validation cohort, and 0.810 (95%CI: 0.744–0.890) in entire cohort. The sensitivity, specificity, PPV, NPV in derivation cohort were 0.667, 0.838, 0.739, and 0.785, respectively. Those in validation cohort were 0.889, 0.718, 0.718, and 0.889, respectively. Those in entire cohort were 0.833, 0.684, 0.664, and 0.846, respectively. The FNR, FPR, MCC, precision, accuracy, F1-score, prevalence, recall were calculated in three cohorts and presented in Supplementary material 6, with high predictive performance. The calibration curves demonstrated close alignment with the reference line (y = x) in three cohorts (F ig. 4G-I).

Third, DCA analysis indicated that implementing the ECMO-IC index (predictive model) generated enhanced net advantages over universal intervention or non-intervention approaches across three cohorts (Fig. 4J-K). RCS regression didn't identify substantial nonlinear relationships between the ECMO-IC index and ECMO-IC (*P* for nonlinearity = 0.396 in derivation cohort; *P* for nonlinearity = 0.087 in validation cohort; *P* for nonlinearity = 0.057 in entire cohort) (Figure S2). However, the odds ratio (OR) of ECMO-IC increased substantially with increasing ECMO-IC index values (*P* for overall < 0.001).

Fourth, to further evaluate the model's reliability, cross-validation analysis was conducted in the entire cohort. The results demonstrated that ROC, PR, calibration curve, and DCA indicated superior predictive capabilities of the ECMO-IC index across 5 distinct cohorts through bootstrap cross-validation and fivefold cross-validation (Figure S3, 4). Additionally, the FNR, FPR, MCC, precision, specificity, accuracy, F1-score, prevalence, recall were applied to assess the ECMO-IC index in 5 diverse cohorts (Supplementary material 7), uncovering that the ECMO-IC index was consistently stable.

### Sensitivity analysis

Sensitivity analysis through excluding participants with any missing data found that the ECMO-IC index exhibited excellent predictive performance, with an AUC of 0.829 (95%CI: 0.756–0.902) in derivation cohort, 0.804 (95%CI: 0.731–0.877) in validation cohort, 0.810 (95%CI: 0.758–0.862) in entire cohort, respectively. The FNR, FPR, MCC, precision, specificity, accuracy, F1-score, prevalence, recall were calculated in three cohorts and presented in Supplementary material 8, with high predictive performance. The predictive and fit performance of the ECMO-IC model before and after data imputation is similar, indicating no significant data leakage.

### Forward-time validation

ECMO-IC was developed and evaluated using a derivation cohort (2015–2020) and a validation cohort (2021–2024) through an innovative LOOCV framework. The AUC values were calculated for individual algorithms and average AUC across both derivation and validation cohorts (Figure S5). We found that the “Lasso + GBM” model ranked sixth based on average AUC values, still demonstrating good predictive performance. However, exhibited substantial AUC values in the derivation group but showed decreased performance in the validation group, indicating overfitting. However, all predictive models exhibited substantial AUC values in the derivation cohort but demonstrated lower performance in the validation cohort, indicating overfitting. Detailed information regarding feature selection, predictive classification, and individual risk scores is provided in Supplementary material 9.

### Multivariable logistic regression, subgroup analysis and interaction effect

In the crude model (without covariate adjustment), univariate logistic regression analysis revealed a notable positive link between the ECMO-IC index and ECMO-IC, with OR and 95% CI values of 1.61 (1.35–1.92) in the derivation cohort, 1.61 (1.35–1.92) in the validation cohort, and 1.61 (1.42–1.82) in the entire cohort (Table 2) (all *P* < 0.001). Upon adjustment for covariates, multivariate logistic regression analysis indicated that the ECMO-IC index remained an independent predictor of ECMO-IC risk, yielding OR and 95% CI values of 2.95 (1.37–6.34) in the derivation cohort, 4.05 (1.55–10.61) in the validation cohort, and 1.84 (1.42–2.38) in the entire cohort (all *P* < 0.05).

**Table 2:** Univariable and multivariable logistic regression analysis for prediction of coagulopathy in patients receiving ECMO

	Derivation cohort		Validation cohort		Entire cohort	
	OR (95% CI)	*P*-value	OR (95% CI)	*P*-value	OR (95% CI)	*P*-value
Coarse model	1.61 (1.35–1.92)	< 0.001*	1.61 (1.36–1.92)	< 0.001*	1.61 (1.42–1.82)	< 0.001*
Model 1	1.88 (1.44–2.44)	< 0.001*	1.74 (1.42–2.13)	< 0.001*	1.60 (1.39–1.84)	< 0.001*
Model 2	2.95 (1.37–6.34)	0.006*	4.05 (1.55–10.61)	0.004*	1.84 (1.42–2.38)	< 0.001*

Coarse model, no covariate was adjusted

Model 1, Sex, age, reasons for ECMO use, ECMO type, DM, HBP, RSD, CSD, HD, RD, NSD, DSD, and other history were adjusted

Model 2, Sex, age, reasons for ECMO use, ECMO type, DM, HBP, RSD, CSD, HD, RD, NSD, DSD, other history, WBC, NEU, Hb, RBC, HCT, LY, NLR, PCT, PDW, ProCT, BNP, Na, Cr, ALT, AST, LDH, ALB, TBIL, DBIL, IBIL, CRP, CK, CKMB, APTT, TT, FIB, first speed, FF, O_2_, AF, PH, PaCO_2_, PaO_2,_ HCO_3_-, OI, HR, RR, SBP, DBP, and MAP were adjusted

*Abbreviations*: *OR* odds ratio, *CI* confidence interval, *ECMO* extracorporeal membrane oxygenation, *DM* diabetes mellitus, *HBP* hypertension, *RSD* respiratory system diseases, *CSD* cardiovascular system diseases, *HD* liver diseases, *RD* renal diseases, *NSD* nervous system diseases, *DSD* digestive system diseases, *HR* heart rate, *SBP* systolic blood pressure, *DBP* diastolic blood pressure, *SI* shock index, *RR* respiratory rate, *SOFA* sequential organ failure assessment, *APACHEII* acute physiology and chronic health evaluation II, *FF* first flow, *AF* air flow, *MAP* mean arterial pressure, *OI* oxygenation index, *WBC* white blood cell, *NEU* neutrophils, *PLT* platelet, *Hb* Hemoglobin, *Cr* creatinine, *ALT* alanine aminotransferase, *Lac* lactate, *AST* aspartate aminotransferase; *LDH* lactate dehydrogenase, *TBIL* total bilirubin, *DBIL* direct bilirubin, *IBIL* indirect bilirubin, *TP* total protein, *ALB* albumin, *PTT* prothrombin time, *APTT* activation partial thromboplastin time, *TT* thrombin time, *FIB* fibrinogen, *ProCT* procalcitonin, *BNP* brain natriuretic peptide, *CRP* C-reactive protein, *CK* creatine kinase, *CK-MB* creatine kinase isoenzymes, *SII* systemic immune-inflammation index, *NLR* neutrophil to lymphocyte ratio, *PLR* platelet to lymphocyte ratio, *HCT* hematocrit, *PCT* plateletocrit *RBC* red blood cell, *RDW* red blood cell volume distribution width, *PDW* platelet distribution width, *LY* lymphocyte. *P < 0.05

Logistic regression analyses were performed across various subgroups to examine potential variations between distinct populations. The findings demonstrated a notable positive link between the ECMO-IC index and ECMO-IC in all subgroups (*P* < 0.05), with exceptions in certain small-sample subgroups (Table 3), suggesting the ECMO-IC model’s reliability. It is worth noting that the ECMO-IC index demonstrates significant predictive value for ECMO-IC in VA-ECMO (OR: 1.63, 95%CI: 1.41–1.87; *P* < 0.001) and VV-ECMO (OR: 1.66, 95%CI: 1.26–2.19; *P* < 0.001). No significant interaction effect was detected between ECMO-IC index and subgroup variables.

**Table 3:** Subgroup analysis for ECMO-IC index in predicting the risk of coagulopathy in patients receiving ECMO

Variable	Count	Percent	OR	Lower	Upper	Adjusted *P* value	*P* for interaction
Hospital							0.638
Shunde	203	76.3	1.65	1.43	1.9	< 0.001	
Nanfang	63	23.7	1.54	1.18	2	0.02	
Outcome							0.177
Survival	107	40.2	1.79	1.38	2.33	< 0.001	
Death	159	59.8	1.46	1.26	1.69	< 0.001	
SOFA							0.494
≤ 9	141	53	1.52	1.28	1.81	< 0.001	
> 9	125	47	1.66	1.38	2	< 0.001	
CRRT							0.143
No	104	39.1	1.78	1.39	2.29	< 0.001	
Yes	162	60.9	1.44	1.24	1.66	< 0.001	
Gender							0.067
Male	184	69.2	1.51	1.31	1.72	< 0.001	
Female	82	30.8	2.05	1.52	2.77	0.001	
Age							0.606
< 50 y	130	48.9	1.6	1.36	1.89	< 0.001	
≥ 50 y	136	51.1	1.72	1.4	2.1	< 0.001	
Reasons							0.091
AM	43	16.2	2.95	1.53	5.67	0.009	
ARDS	31	11.7	1.58	1.1	2.26	0.086	
CPR	25	9.4	1.5	0.95	2.38	0.18	
DM	3	1.1	NA	NA	NA	NA	
ICM	8	3	NA	NA	NA	NA	
MI	90	33.8	1.5	1.22	1.85	0.001	
PTE	9	3.4	1.19	0.54	2.64	0.659	
SP	18	6.8	1.66	0.9	3.04	0.102	
VHD	3	1.1	NA	NA	NA	NA	
MTT	6	2.3	2.7	0.27	26.6	0.395	
SA	4	1.5	1.45	0.57	3.7	0.434	
Other	26	9.8	1.7	1.14	2.52	0.029	
ECMO type							0.061
VA	203	76.3	1.63	1.41	1.87	< 0.001	
VV	60	22.6	1.66	1.26	2.19	0.004	
VAV	3	1.1	0	0	Inf	1	
Ever experienced CPR							0.155
No	101	38	1.98	1.49	2.64	< 0.001	
Yes	165	62	1.57	1.35	1.81	< 0.001	
Hospital LOS							0.436
≤ 16	128	48.1	1.68	1.4	2.02	< 0.001	
> 16	138	51.9	1.52	1.27	1.81	< 0.001	
ICU LOS							0.141
≤ 9	123	46.2	1.79	1.46	2.19	< 0.001	
> 9	143	53.8	1.47	1.26	1.73	< 0.001	
MVT							0.072
≤ 7	124	46.6	1.87	1.52	2.3	< 0.001	
> 7	142	53.4	1.43	1.22	1.67	< 0.001	
ECMOT							0.137
≤ 4	121	45.5	1.79	1.47	2.17	< 0.001	
> 4	145	54.5	1.47	1.25	1.73	< 0.001	
DM							0.316
No	212	79.7	1.56	1.37	1.78	< 0.001	
Yes	54	20.3	1.87	1.34	2.61	0.003	
HBP							0.825
No	189	71.1	1.6	1.39	1.85	< 0.001	
Yes	77	28.9	1.66	1.28	2.15	0.002	
RSD							0.124
No	260	97.7	1.64	1.45	1.87	< 0.001	
Yes	6	2.3	1	0.54	1.86	0.996	
CSD							0.989
No	245	92.1	1.57	1.38	1.78	< 0.001	
Yes	21	7.9	NA	NA	NA	NA	
HD							0.985
No	258	97	1.59	1.4	1.8	< 0.001	
Yes	8	3	NA	NA	NA	NA	
RD							0.834
No	253	95.1	1.62	1.43	1.84	< 0.001	
Yes	13	4.9	1.5	0.73	3.1	0.275	
NSD							0.295
No	256	96.2	1.63	1.43	1.85	< 0.001	
Yes	10	3.8	1.16	0.62	2.16	0.644	
DSD							0.984
No	262	98.5	1.6	1.42	1.82	< 0.001	
Yes	4	1.5	NA	NA	NA	NA	
Protein							0.575
No	42	15.8	1.52	1.14	2.03	0.04	
Yes	224	84.2	1.67	1.45	1.92	< 0.001	
VAD use (≥ 2)							0.113
No	114	42.9	1.87	1.47	2.37	< 0.001	
Yes	152	57.1	1.49	1.29	1.73	< 0.001	

*Abbreviations*: *ECMO* extracorporeal membrane oxygenation, *CRRT* continuous renal replacement therapy, *VA* venoarterial, *VV* venovenous, *VAV* venoarterial-venous, *DM* diabetes mellitus, *HBP* hypertension, *RSD* respiratory system diseases, *CSD* cardiovascular system diseases, *HD* liver diseases, *RD* renal diseases, *NSD* nervous system diseases, *DSD* digestive system diseases, *AM* acute myocarditis, *ARDS* acute respiratory distress syndrome, *CPR* cardiopulmonary resuscitation, *DM* dermatomyositis, *ICM* cardiomyopathy, *MI* myocardial infarction, *PTE* pulmonary embolism, *SP* severe pneumonia, *VHD* heart valve disease, *MTT* multiple trauma, *SA* surgical assistance, *ICU* intensive care unite, *LOS* length of stay, *MVT* mechanical ventilation time, *ECMOT* extracorporeal membrane oxygenation time, *VAD* vasoactive drugs

### Comparison of optimised ECMO-IC index to APACHE II and SOFA

**Fig. 5:**
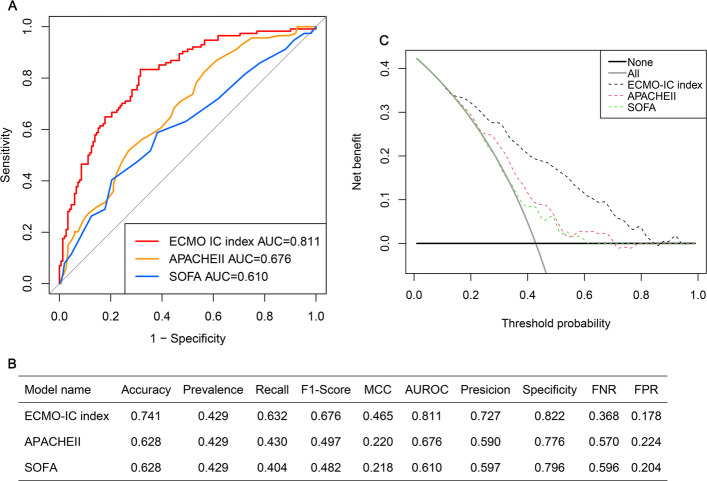
Comparison of optimized ECMO-IC index to APACHE II and sequential organ failure assessment (SOFA). **A** Multiple ROC analysis was performed to compare the diagnostic performance of the ECMO-IC index against APACHE II and SOFA. **B** The model’s predictive performance was compared through a comprehensive array of metrics. **C**: Decision curve analysis was applied to evaluate the clinical usefulness of ECMO-IC index against APACHE II and SOFA. The Y-axis represents the net benefit. The black line represents the hypothesis that no patients die. The Xaxis represents the threshold probability. The threshold probability is where the expected benefit of treatment is equal to the expected benefit of avoiding treatment

Multiple ROC analyses revealed that the ECMO-IC index demonstrated superior performance in predicting ECMO-IC compared with APACHE II (AUC = 0.676, 95%CI: 0.642–0.710) and SOFA (AUC = 0.610, 95%CI: 0.552–0.668) (Fig. 5A) (*P* < 0.05). Furthermore, the FNR, FPR, MCC, precision, specificity, accuracy, F1-score, prevalence, recall were superior to APACHE II and SOFA (Fig. 5B). Remarkably, the DCA plot illustrated that the ECMO-IC index displayed enhanced performance relative to the APACHE II and SOFA scores (Fig. 5C).

### Model interpretation

**Fig. 6:**
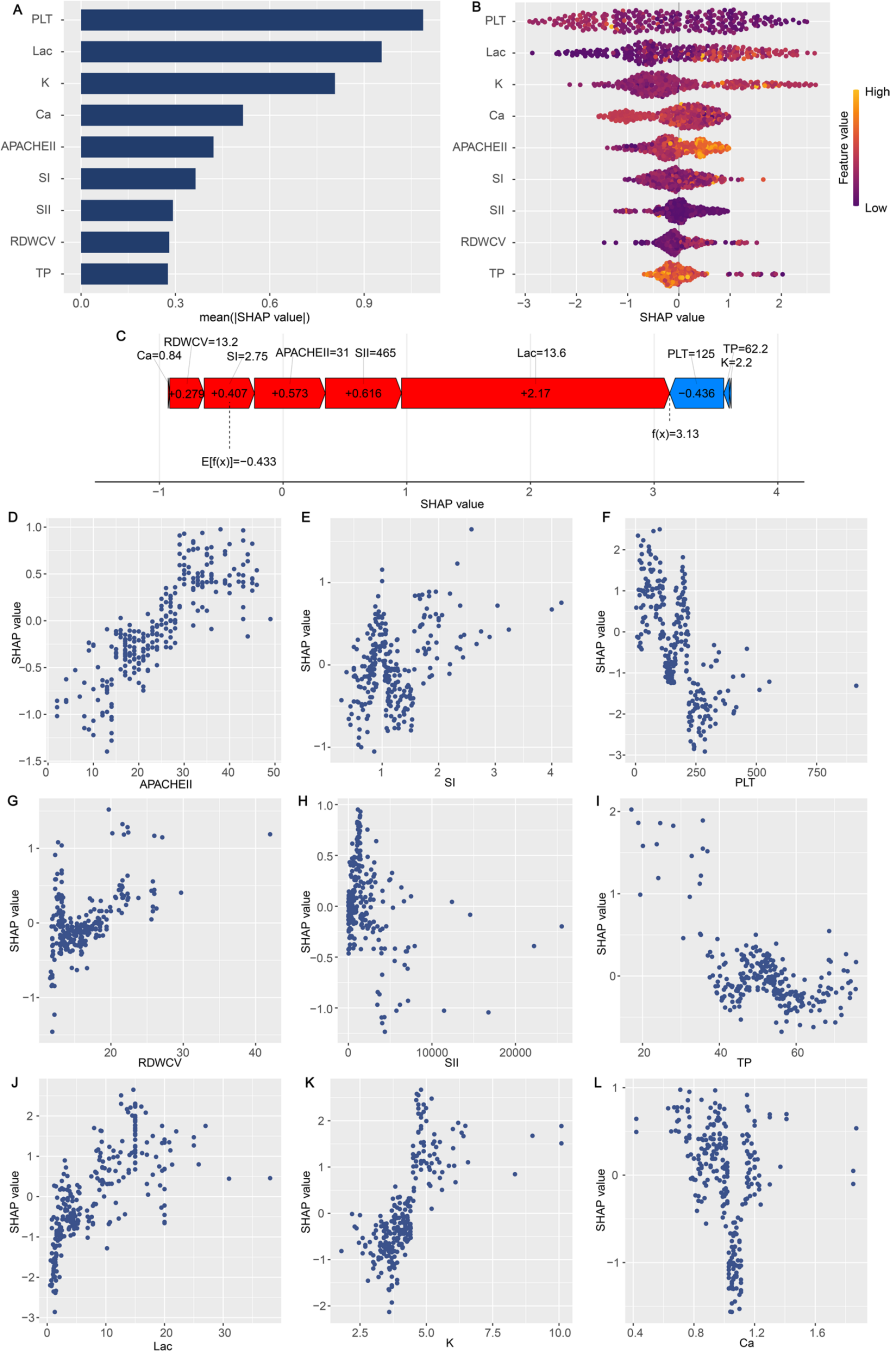
Global and local explanation by the Shapley Additive Explanations (SHAP) method. **A** Summary plot showed the 9 features ranking by mean absolute SHAP values. **B** Global model explanation. Each variable name is shown on the left-hand side with the variable with the greatest contribution listed at the top. **C** An example of risk factor analysis for a patient with ECMO-IC which represented the individual ECMO towards the “coagulopathy” class. (D-L) One-way SHAP dependence plot of the 9 important predictors. **D** APACHE II; (**E**) SI; (**F**) PLT; (**G**) RDWCV; (**H**) SII; (**I**) TP; (**J**) Lactate; (**K**) K; (**L**) Ca. Each dependence plot shows how a single feature affects the output of the prediction model, and each dot represents a single patient. PLT: platelet; SII: systemic immune-inflammation index; RDWCV: red blood cell volume distribution width; SI: shock index; TP: total protein; APACHE II: acute physiology and chronic health evaluation II

The SHAP algorithm was utilized to elucidate the predictive significance of the selected variables in the optimal model for ECMO-IC. Figure 6A displays the comparative significance and impact of the 9 features in the ECMO-IC model, derived through SHAP algorithm interpretation of the ECMO-IC model predictions. As shown in Fig. 6A, it can be observed that the top 5 variables are PLT, lactate, K, Ca and APACHE II.

Global explanations: Fig. 6B visually demonstrates the range and distribution of the 9 characteristics with respect to model performance. The positively related features were as follows: Lactate, K and APACHE II (higher values of these parameters corresponded to increased probability of coagulopathy development following ECMO); conversely, PLT, Ca, and TP demonstrated negative correlations. Significantly, these features can influence predictions in opposite directions (elevating or reducing ECMO-IC) for patients with varying characteristics, although the impact of specific feature values on predictions remains constant.

Local explanations: To better understand the optimized ECMO-IC model’s predictions, patient-specific risk assessments and their associated factors underwent analysis through SHAP values. In the case of the ECMO-IC patient exhibiting the maximum predicted SHAP value (3.13), lactate (13.6 mmol/L, SHAP value = 2.17), APACHE II (31, SHAP value = 0.573), and SI (2.75, SHAP value = 0.407) emerged as principal elements contributing to the heightened SHAP score (Fig. 6C).

Finally, the partial dependence plots demonstrated the comprehensive correlation between feature and risk distribution. These visualizations revealed the linear or nonlinear connections and trending patterns between PLT, lactate, SII, K, TP, SI, RDWCV, APACHE II, Ca and SHAP value (Fig. 6D-L).

### Logistic regression, RCS regression and threshold effect analysis

**Fig. 7:**
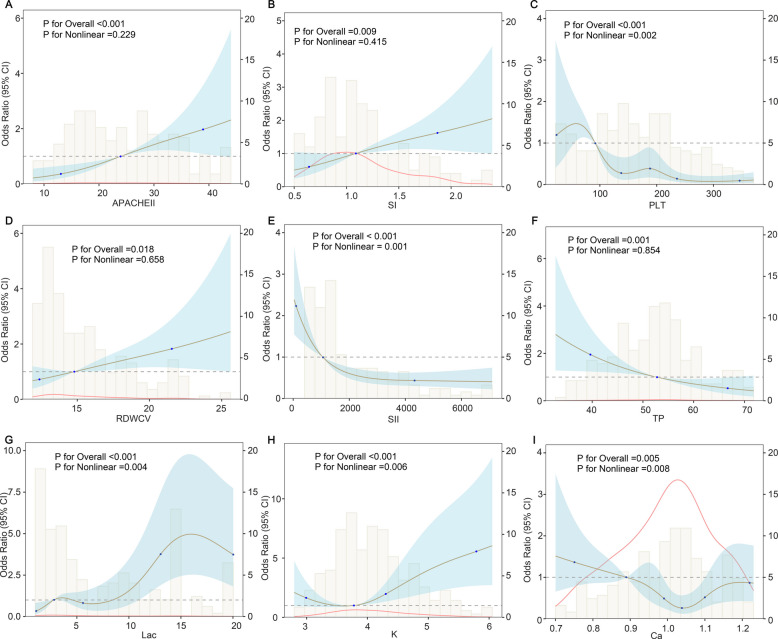
Potential nonlinear association for the levels of 9 predictors with ECMO-IC risk measured by restricted cubic spline regression with optimal knots. **A** APACHE II. **B** SI. **C** PLT. **D** RDWCV. **E** SII. **F** TP. **G** Lactate. **H** K. **I** Ca. The brown line and shadow area represent the estimated OR and the 95% CI. PLT: platelet; SII: systemic immune-inflammation index; RDW: red blood cell volume distribution width; SI: shock index; TP: total protein; APACHE II: acute physiology and chronic health evaluation II

Additional analysis of feature significance in the predictive model involved univariable logistic and RCS regression to examine linear and nonlinear correlations. Supplementary material 10 provided optimised knot for RCS analysis. The findings revealed 5 and 8 predictors with nonlinear and linear correlations to ECMO-IC risk (all *P* < 0.008), respectively (Table 4), with results illustrated in Fig. 7. PLT (*P* for nonlinearity = 0.002), SII (*P* for nonlinearity = 0.001), K (*P* for nonlinearity = 0.006), Ca (*P* for nonlinearity = 0.008), and lactate (*P* for nonlinearity = 0.004) revealed significant nonlinear relationships with ECMO-IC risk (Table 4 and Fig. 7). However, APACHE II (*P* for nonlinearity = 0.229), SI (*P* for nonlinearity = 0.415), RDWCV (*P* for nonlinearity = 0.657), and TP (*P* for nonlinearity = 0.854) demonstrated significant linear relationships with ECMO-IC risk but lacked evidence of significant nonlinear associations.

**Table 4:** Nonlinear and linear associations between clinical variables and coagulopathy risk in patients receiving ECMO

Factors	Linear association		Nonlinear association	
	OR (95%CI)	*P*	Chi-square	*P*
APACHEII	1.07(1.04–1.10)	< 0.001*	1.450	0.229
SI	2.00(1.25–3.17)	0.004*	0.664	0.415
PLT	0.993(0.990–0.996)	0.003*	17.279	0.002*
RDWCV	1.10(1.03–1.18)	0.006*	0.196	0.657
SII	0.99(0.99–0.99)	0.003*	10.47	0.001*
K	1.64(1.22–2.19)	0.001*	10.308	0.006*
Ca	0.24(0.06–1.07)	0.061	13.660	0.008*
TP	0.95(0.93–0.98)	< 0.001*	0.034	0.854
Lac	1.11(1.06–1.15)	< 0.001*	13.289	0.004*

*Abbreviations*: *ECMO* extracorporeal membrane oxygenation, *APACHEII* acute physiology and chronic health evaluation II, *SI* shock index, *PLT* platelet, *TP* total protein, *SII* systemic immune-inflammation index, *RDW* red blood cell volume distribution width, *Lac* lactate; *P < 0.05

Additionally, threshold effect analysis with generalized additive linear model was conducted to yield inflection point for PLT (95 × 10^9^/L), lactate (5.7 mmol/L), SII (200), K (4.4 mmol/L), TP (45.6 g/L), SI (0.8), RDWCV (14%), APACHE II (15), Ca (1.03 mmol/L). Specifically, ECMO-IC risk exhibited a notable increase when measurements of lactate (OR = 1.27, 95%CI = 1.06–1.52; *P* = 0.010), K (OR = 2.23, 95%CI = 1.20–4,17; *P* = 0.012), SI (OR = 1.70, 95%CI = 1.02–2.83; *P* = 0.041), RDWCV (OR = 1.09, 95%CI = 1.00–1.18; *P* = 0.045), APACHE II (OR = 1.05, 95%CI = 1.02–1.09; *P* = 0.001) exceeded specific threshold values. Or when PLT (OR = 0.995, 95%CI = 0.991–0.999; *P* = 0.008), SII (OR = 0.999, 95%CI = 0.999–0.999; *P* = 0.020), TP (OR = 0.96, 95%CI = 0.93–1.00; *P* = 0.028) and Ca (OR = 0.013, 95%CI = 0.001–0.172; *P* = 0.001) levels fell below certain cutoff points, they no longer provide protection against the ECMO-IC risk. Detailed information on threshold effect analysis was provided in Supplementary material 11.

The RCS evaluation revealed a U-shaped correlation between K levels and ECMO-IC risk, suggesting that K levels at either extreme contribute to ECMO-IC development. Compared to K levels between 3 and 4.4, when blood K is below 3 (OR = 4.94, 95%CI = 1.46–16.73; *P* = 0.01) or above 4.4 (OR = 4.31, 95%CI = 2.42–7.66; *P* < 0.001), there is a substantial elevation in ECMO-IC risk.

### Clinical availability of the ECMO-IC model

Given the accessibility of the 9 predictive features, we implemented a web-based calculator to facilitate point-of-care clinical decision support in ECMO-IC management. The online calculator is freely accessible at https://genglongliu.shinyapps.io/DynNomapp/.

## Discussion

In recent years, against the backdrop of the COVID-19 pandemic, the global use of ECMO has surged. ECMO-IC is an important cause of death in ECMO patients, currently, ECMO-IC lacks an effective ML model to early identify coagulopathy risk and guide individualized intervention to improve ECMO survival. This study utilized two centers with 266 patients undergoing ECMO to construct and internally validate a reliable, generalizable, and interpretable ECMO-IC model for ECMO patients based on routine clinical parameters (PLT, lactate, SII, K, TP, SI, RDWCV, APACHE II, and Ca) prior to initiation of ECMO support within 24 h. The resulting ECMO-IC model offers a readily available, interpretable, and accurate diagnostic approach for assessing coagulopathy risk following ECMO, with promising clinical utility. Early warning and timely intervention with these abnormal and modifiable predictors could prevent the occurrence of coagulopathy in ECMO patients.

This multicenter cohort study revealed that coagulopathy rates during ECMO reached 43.35% in Shunde Hospital, 41.27% in Nanfang Hospital, 40.80% in derivation cohort and 44.68% in validation cohort. These findings align with prior research demonstrating coagulopathy prevalence (manifesting as thrombosis or severe bleeding) in ECMO cases varying between 37 and 62% [9, 24]. We also found the ECMO-IC was markedly correlated to higher mortality and CRRT/albumin/VAD (≥ 2) usage. Coagulation disorders frequently occurred in the EOLIA trial (Even in a controlled setting), nearly half (46%) of patients randomly assigned to ECMO required blood transfusions due to bleeding [25]. The pathophysiology of these complications during ECMO is complex, dynamic and not fully understood [26]. This may explain why standard methods for monitoring coagulation are imperfect and studies using traditional biostatistical methods have been unable to consistently identify common risk factors. Therefore, an effective ECMO-IC prediction model with the help of ML will be of great clinical importance in this age of precision medicine.

The Boruta algorithm [19, 22, 27] was utilized to examine over 70 pathophysiological parameters, aiming to streamline redundant factors and establish a more precise, simplified variable set for the ECMO-IC model’s practical implementation. A recent bleeding risk model involving 47 variables lacks feature selection, which makes it difficult to use [14]. Through Boruta algorithm-based variable evaluation, 17 distinct parameters emerged as essential components for constructing the ECMO-IC model. The novel LOOCV framework markedly enhanced the model’s transferability and reliability. The study incorporated two critical care centers containing substantial ECMO cases to strengthen statistical validity and ensure model precision. Previous research typically relied on modeling algorithms selected based on individual researcher expertise and preferences [13, 14]. To overcome this constraint, the research employed 12 ML algorithms appropriate for constructing an improved diagnostic model. These algorithms generated 105 combinations. The ML technique has been characterized as a “black-box” approach with limited transparency regarding how predictions are generated [28]. The SHAP methodology [21, 22] was implemented to elucidate the “black-box” nature of ECMO-IC models in predicting coagulopathy during ECMO. This approach provides both comprehensive model functionality analysis and specific prediction explanations for individual ECMO-IC risk assessments using patient-specific data. SHAP analysis revealed that PLT, lactate, K, Ca and APACHE II ranked as the most significant parameters in the ECMO-IC model. These findings align with the initial Boruta algorithm feature selection results, highlighting PLT, lactate, and K as crucial variables. However, SHAP values have known limitations that are not acknowledged here [29]. First, SHAP feature attributions can be unstable and misleading in the presence of correlated features, such as PLT and SOFA in our model. Second, SHAP may arbitrarily distribute importance among collinear features, and small changes in data or model can significantly alter the SHAP importance rankings. Therefore, clinicians can use SHAP values to interpret the model, but we need to be cautious when using the implications of these SHAP values in real-world. Reassuringly, an online computing platform was established based on the optimal ECMO-IC model for the early diagnosis and risk assessment of coagulopathy in ECMO patients. Such a patient-level interpretable model is free-to-use by clinicians, enabling them to combine it with their experiential knowledge to facilitate decision-making. Notably, if a patient presents with a high risk of ECMO-IC requiring anticoagulation therapy, close monitoring of anticoagulation parameters (AT/AT-III, ACT, etc.) [30] is crucial for initiating ECMO anticoagulation management and evaluating its efficacy.

Several studies have reported on the use of machine learning to predict risk factors for bleeding and thrombosis during ECMO [13, 14]. Abbasi et al. [13] included 44 consecutive patients supported with ECMO using five ML models (RF, recursive feature elimination, decision trees, k-nearest neighbors and logistic regression) to predict hemorrhage and thrombosis risk. The predictive efficacy of the models is relatively inferiority, AUC values were around 0.6. Kamio et al. [14] conducted a retrospective cohort study including 357 ECMO patients using four ML models (RF, SVM, XGBoost, and GBM) to predict bleeding risk. Ultimately, 47 variables were incorporated, yielding a predicted AUC value of approximately 0.7. Furthermore, these two studies did not provide the timing of variable acquisition and lacked advanced interpretability methods to explain model. Our study, including 266 ECMO patients, comprehensively assesses routine clinical characteristics collected before ECMO support initiation within 24 h to develop a reliable, accurate, and explainable ML model for estimating ECMO-IC risk and to identify modifiable factors. The model demonstrated superior diagnostic capabilities, achieving a mean AUC of 0.815 across both cohorts, along with notable discriminatory power, model fit, and clinical utility.

SI is defined as the ratio of HR to SBP, which has been demonstrated to be linked to indices of end-organ perfusion. SI levels have been found to be a key factor in early triggering coagulopathy in trauma patients, with a risk of coagulopathy six times higher than in patients with low SI levels [31]. Logistic and RCS regression uncover that SI is a notable linear association with ECMO-IC risk, when SI ≥ 0.8, the ECMO-IC risk was markedly higher. Lactate is a reliable indicator of tissue perfusion deficiency. A large body of evidence proposes that lactate and lactate clearance as risk factor for patients receiving ECMO [32, 33]. Serum lactate levels and lactate clearance at 24 h represented the most powerful independent predictors of short-term survival in ECMO patients [32]. Our study found that elevated lactate correlates positively with ECMO-IC risk, especially when lactate levels exceed 5.7 mmol/L. SII is a novel parameter based on lymphocyte, neutrophil, and platelet counts, which can reflect a patient’s systemic inflammatory status and local immune response. Jiang et al. [34] and Uleng et al. [35] uncovered that low SII values were associated with an elevated mortality risk from sepsis within 28 days. Low SII results, due to low PLT and neutrophil counts, may indicate significant inflammation or bone marrow suppression. The commencement of ECMO correlates with an immediate and complex inflammatory response, comparable to that seen in systemic inflammatory response syndrome [36], including sepsis. Our study showed that low SII (less than 200) was positively correlated with ECMO-IC risk, which was most likely due to low PLT counts. RDW levels reflect red blood cell size heterogeneity and indicate a body's response to oxidative stress and inflammation [37, 38]. RDW can serve as a simple and inexpensive biomarker for predicting mortality and acute kidney injury incidence in ECMO patients [37, 38]. Our study revealed a notable positive link between the RDW and ECMO-IC risk, especially when RDW exceeds 14%. Patients receiving ECMO support are in a state of high protein catabolism and are prone to acquired malnutrition, which affects outcomes [39]. Total protein represents the body's protein level, and low TP (above 45.6 g/L) was significantly associated with ECMO-IC risk. Ionized Ca ions play an important role in maintaining hemodynamics in pediatric and neonatal patients undergoing ECMO treatment, and early intervention for hypocalcemia may be beneficial to patients [40, 41]. However, the definition of hypocalcaemia and the timing of intervention remain unclear. In this study, ionized Ca levels above 1.03 mmol/L were significantly associated with the risk of adult ECMO-IC. It is worth noting that both high K (> 4.4 mmol/L) and low K (< 3 mmol/L) levels can result in the occurrence of ECMO-IC for the first time. This finding might be explained by several mechanisms: Abnormal blood K levels can 1) inhibit platelet activity and function; 2) interfere with coagulation factor activity or synthesis; 3) damage vascular endothelial cell function and weaken vascular smooth muscle contraction [42]. These predictive indicators prior to initiation of ECMO support within 24 h reflect different pathophysiological states of the disease and help predict the risk of ECMO-IC. Inflection point of modifiable predictors generated through threshold effect analysis may influence several clinical actions, such as when PLT < 95 × 10^9^/L or SII < 200, this warns the need for empirical blood transfusion; when Ca < 1.03 mmol/L or K < 3 mmol/L, this warns the requirement for electrolyte replacement; when SI ≥ 0.8 or lactate ≥ 5.7 mmol/L, this cues the need for enhancing circulation; when TP < 45.6 g/L, this cues the requirement for nutritional support. As these findings have not yet been implemented in clinical practice, we are unable to draw a clear utility. It is necessary to validate these findings in future clinical studies.

In contrast to previously published studies, this research presents several key distinctions. (1) For the first time, a readily available, precise, and explainable prediction model for coagulopathy in ECMO patients based on routine clinical characteristics was developed in two cohorts with larger sample sizes. It may provide significant benefits in clinical practice by enabling resource planning and timely intervention, as the preparation and administration of blood products can be time-consuming. (2) By integrating Boruta’s feature selection algorithm with 12 machine learning algorithms, a total of 105 model combinations were created to construct consensus prediction models capable of identifying critical feature variables, optimizing performance, and minimizing overfitting risks. (3) The SHAP approach was employed to address concerns regarding the “black-box” nature of ML by offering feature importance rankings and visualizing both global and individual risk predictions. This patient-specific interpretable model enables clinicians to integrate predictive outcomes with their clinical judgment, thereby supporting decision-making. (4) RCS regression and threshold effect analysis illuminate nonlinear associations between ECMO-IC and predictor, and provides inflection point for predictor. Clinicians can administer corresponding interventions in a timely manner based on the thresholds of the predictors to reduce the incidence of coagulopathy during ECMO.

### Limitations

Despite meticulous and thorough research efforts, several constraints merit acknowledgment. Initially, although the ECMO-IC model exhibited remarkable predictive capabilities, as a retrospective predictive model, the present model remains internally validated only, and the sample size of the current predictive model remains insufficient. These may lead to model overfitting and diminished generalisation capability. The present model requires validation using a prospective, large-scale external dataset before implementation in general clinical practice. Second, the study maybe suffer from forms of data leakage, such as feature selection based on three cohorts intersection, model selection based on average AUC, MICE in entire cohorts, which may cause potential information leakage from the derivation cohort into the validation cohort, resulting in over-optimistic results. To ensure that valuable clinical variables are retained as much as possible, we selected the variables based on intersection of three cohorts. The final model (Lasso + LDA) was chosen based on average AUC of both cohorts, considering model fit and simplicity. In terms of multiple interpolation methods to address missing values in the entire dataset, the sensitivity results show negligible bias. In the future, when verifying this model, it is necessary to avoid such data leakage. Third, recently, an updated SOFA-2 [43] framework has been proposed and explicitly incorporates ECMO. Given the extended timeframe of our study (2015–2024), which includes some patients from considerable time ago, certain detailed indicators within the SOFA-2 score (such as vasoactive drug usage and delirium) proved challenging to collect. Future research should compare the current model with the SOFA-2. Fourth, the predictive model used in this study relies on static data and can only provide risk predictions at a single point in time, failing to take into account changes in the course of the disease. Given the complexity and dynamic nature of patients receiving ECMO treatment, a dynamic predictive model needs to be developed in the future. Fifth, there are currently no specific diagnostic guidelines for ECMO-IC, so we used the ISTH SSC diagnosis for coagulopathy. This may lead to some missed diagnoses and misdiagnoses. In the future, our results need to be validated in a cohort of confirmed ECMO-IC according to specific diagnostic guidelines for ECMO-IC, or we redevelop new models for ECMO-IC. Sixth, all predictive variables were collected within 24 h before ECMO initiation; future studies may consider incorporating values obtained during ECMO or the changes/trends in these values to determine whether this would further enhance the predictive performance of the current model. Seventh, threshold effect analysis with generalized additive linear model offer inflection point for each predictor. This evidence should be presented as hypothesis-generating, not as recommendations for intervention. Clinicians must consider all of these factors comprehensively to ensure accurate intervention measures.

## Conclusion

This study established and internally validated a consensus diagnostic framework (ECMO-IC index) to forecast coagulopathy following ECMO. The ECMO-IC index represents a readily available, comprehensible, and precise instrument for identifying early ECMO-IC risk, offering practical clinical utility. Timely intervention with these abnormal and modifiable predictors may help to prevent the development of coagulopathies.

## Supplementary Information

Supplementary Material 1. Figure S1. A total of 105 ML algorithm combinations of prediction models using the LOOCV framework and further calculated the area under curve (AUC) of each model in derivation and validation cohorts. 

Supplementary Material 2. Figure S2. Potential nonlinear for the levels of ECMO-IC index with ECMO-IC risk measured by restricted cubic spline regression with optimal knots. (A) Derivation cohort; (B)Validation cohort; (C) Entire cohort. The brown line and shadow area represent the estimated OR and the 95% CI.

Supplementary Material 3. FigureS3. 5-fold and Bootstrap cross-validation applied to evaluate the reliability and stability ability of ECMO-IC index. (A, B) ROC analysis through 5-fold (A) and Bootstrap (B) cross-validation. (C, D) PR analysis through 5-fold (C) and Bootstrap (D) cross-validation.

Supplementary Material 4. FigureS4. 5-fold and Bootstrap cross-validation applied to evaluate the reliability and stability ability of ECMO-IC index. (A, B) Calibration curves analysis through 5-fold (A) and Bootstrap (B) cross-validation. (C, D) DCA analysis through 5-fold (C) and Bootstrap (D) cross-validation.

Supplementary Material 5. 

Supplementary Material 6. 

Supplementary Material 7.

Supplementary Material 8.

Supplementary Material 9.

Supplementary Material 10.

Supplementary Material 11.

Supplementary Materrial 12 

Supplementary Material 13.

Supplementary Material 14.

Supplementary Material 15.

Supplementary Material 16.

Supplementary Material 17.

Supplementary Material 18.

## Data Availability

The original data presented in the study are included in the article/Supplementary Material. Further inquiries can be directed to the corresponding author. Executable code was provided in Supplementary material 12.

## Abbreviation

ECMO-IC: Extracorporeal membrane oxygenation-induced coagulopathy
ICU: Intensive care unit
ML: Machine learning
RCS: Restricted cubic spline
SHAP: Shapley Additive Explanations
CI: Confidence interval
OR: Odds ratio
SE: Standard error;
ROC: Receiver operating characteristic
AUC: Area under curve
Enet: Elastic network
LASSO: Least absolute shrinkage and selection operator
RF: Random forest
GBM: Gradient boosting machine
SuperPC: Supervised principal components
plsRglm: Partial least squares regression for generalized linear models, SVM: support vector machine
LOOCV: Leave-one-out cross-validation
DCA: Decision curve analysis
LDA: Linear discriminant analysis
XGBoost: EXtreme Gradient Boosting
GBM: Gradient boosting machine
TRIPOD: Transparent Reporting of a Multivariable Prediction Model forIndividual Prognosis or Diagnosis
MCC: Matthews correlation coefficient
FNR: False negative rate
FPR: False positive rate
SD: Standard deviation
ECMO: Extracorporeal membrane oxygenation
CRRT: Continuous renal replacement therapy
VA: Venoarterial
VV: Venovenous
VAV: Venoarterial-venous
PLT: Platelet
SII: Systemic immune-inflammation index
RDW: Red blood cell volume distribution width
SI: Shock index
TP: Total protein
APACHE II: Acute physiology and chronic health evaluation II
