# Supplementary material for: Development and internal validation of an interpretable machine learning model to predict coagulopathy following extracorporeal membrane oxygenation: a retrospective multicenter study
Source: Scand J Trauma Resusc Emerg Med. 2026 Jan 28;34:45. doi: 10.1186/s13049-026-01564-x (PMC12924354; doi:10.1186/s13049-026-01564-x)
Supplement: Supplementary file 1 — Supplementary Material 1. Figure S1. A total of 105 ML algorithm combinations of prediction models using the LOOCV framework and further calculated the area under curve (AUC) of each model in derivation and validation cohorts. [file 13049_2026_1564_MOESM1_ESM.pdf]

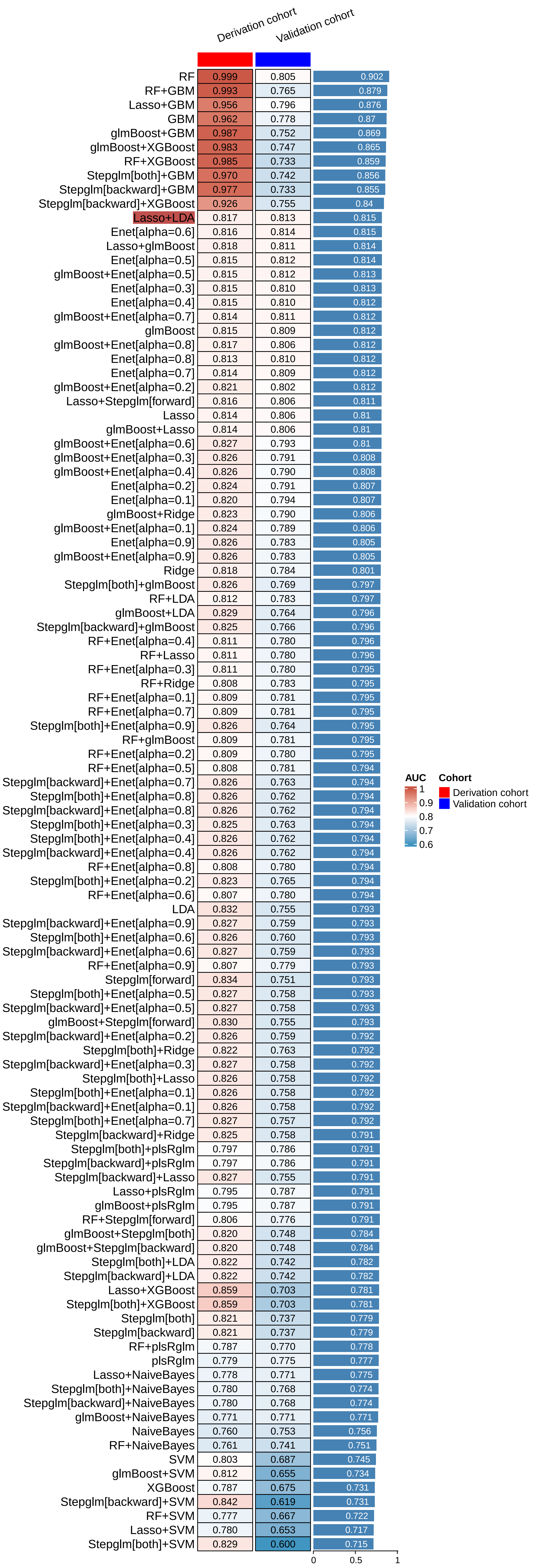
Derivation cohort
Validation cohort
RF 0.999 0.805 0.902
RF+GBM 0.993 0.765 0.879
Lasso+GBM 0.956 0.796 0.876
GBM 0.962 0.778 0.87
glmBoost+GBM 0.987 0.752 0.869
glmBoost+XGBoost 0.983 0.747 0.865
RF+XGBoost 0.985 0.733 0.859
Stepglm[both]+GBM 0.970 0.742 0.856
Stepglm[backward]+GBM 0.977 0.733 0.855
Stepglm[backward]+XGBoost 0.926 0.755 0.84
Lasso+LDA 0.817 0.813 0.815
Enet[alpha=0.6] 0.816 0.814 0.815
Lasso+glmBoost 0.818 0.811 0.814
Enet[alpha=0.5] 0.815 0.812 0.814
glmBoost+Enet[alpha=0.5] 0.815 0.812 0.813
Enet[alpha=0.3] 0.815 0.810 0.813
Enet[alpha=0.4] 0.815 0.810 0.812
glmBoost+Enet[alpha=0.7] 0.814 0.811 0.812
glmBoost 0.815 0.809 0.812
glmBoost+Enet[alpha=0.8] 0.817 0.806 0.812
Enet[alpha=0.8] 0.813 0.810 0.812
Enet[alpha=0.7] 0.814 0.809 0.812
glmBoost+Enet[alpha=0.2] 0.821 0.802 0.812
Lasso+Stepglm[forward] 0.816 0.806 0.811
Lasso 0.814 0.806 0.81
glmBoost+Lasso 0.814 0.806 0.81
glmBoost+Enet[alpha=0.6] 0.827 0.793 0.81
glmBoost+Enet[alpha=0.3] 0.826 0.791 0.808
glmBoost+Enet[alpha=0.4] 0.826 0.790 0.808
Enet[alpha=0.2] 0.824 0.791 0.807
Enet[alpha=0.1] 0.820 0.794 0.807
glmBoost+Ridge 0.823 0.790 0.806
glmBoost+Enet[alpha=0.1] 0.824 0.789 0.806
Enet[alpha=0.9] 0.826 0.783 0.805
glmBoost+Enet[alpha=0.9] 0.826 0.783 0.805
Ridge 0.818 0.784 0.801
Stepglm[both]+glmBoost 0.826 0.769 0.797
RF+LDA 0.812 0.783 0.797
glmBoost+LDA 0.829 0.764 0.796
Stepglm[backward]+glmBoost 0.825 0.766 0.796
RF+Enet[alpha=0.4] 0.811 0.780 0.796
RF+Lasso 0.811 0.780 0.796
RF+Enet[alpha=0.3] 0.811 0.780 0.795
RF+Ridge 0.808 0.783 0.795
RF+Enet[alpha=0.1] 0.809 0.781 0.795
RF+Enet[alpha=0.7] 0.809 0.781 0.795
Stepglm[both]+Enet[alpha=0.9] 0.826 0.764 0.795
RF+glmBoost 0.809 0.781 0.795
RF+Enet[alpha=0.2] 0.809 0.780 0.795
RF+Enet[alpha=0.5] 0.808 0.781 0.794
Stepglm[backward]+Enet[alpha=0.7] 0.826 0.763 0.794
Stepglm[both]+Enet[alpha=0.8] 0.826 0.762 0.794
Stepglm[backward]+Enet[alpha=0.8] 0.826 0.762 0.794
Stepglm[both]+Enet[alpha=0.3] 0.825 0.763 0.794
Stepglm[both]+Enet[alpha=0.4] 0.826 0.762 0.794
Stepglm[backward]+Enet[alpha=0.4] 0.826 0.762 0.794
RF+Enet[alpha=0.8] 0.808 0.780 0.794
Stepglm[both]+Enet[alpha=0.2] 0.823 0.765 0.794
RF+Enet[alpha=0.6] 0.807 0.780 0.794
LDA 0.832 0.755 0.793
Stepglm[backward]+Enet[alpha=0.9] 0.827 0.759 0.793
Stepglm[both]+Enet[alpha=0.6] 0.826 0.760 0.793
Stepglm[backward]+Enet[alpha=0.6] 0.827 0.759 0.793
RF+Enet[alpha=0.9] 0.807 0.779 0.793
Stepglm[forward] 0.834 0.751 0.793
Stepglm[both]+Enet[alpha=0.5] 0.827 0.758 0.793
Stepglm[backward]+Enet[alpha=0.5] 0.827 0.758 0.793
glmBoost+Stepglm[forward] 0.830 0.755 0.793
Stepglm[backward]+Enet[alpha=0.2] 0.826 0.759 0.792
Stepglm[both]+Ridge 0.822 0.763 0.792
Stepglm[backward]+Enet[alpha=0.3] 0.827 0.758 0.792
Stepglm[both]+Lasso 0.826 0.758 0.792
Stepglm[both]+Enet[alpha=0.1] 0.826 0.758 0.792
Stepglm[backward]+Enet[alpha=0.1] 0.826 0.758 0.792
Stepglm[both]+Enet[alpha=0.7] 0.827 0.757 0.792
Stepglm[backward]+Ridge 0.825 0.758 0.791
Stepglm[both]+plsRglm 0.797 0.786 0.791
Stepglm[backward]+plsRglm 0.797 0.786 0.791
Stepglm[backward]+Lasso 0.827 0.755 0.791
Lasso+plsRglm 0.795 0.787 0.791
glmBoost+plsRglm 0.795 0.787 0.791
RF+Stepglm[forward] 0.806 0.776 0.791
glmBoost+Stepglm[both] 0.820 0.748 0.784
glmBoost+Stepglm[backward] 0.820 0.748 0.784
Stepglm[both]+LDA 0.822 0.742 0.782
Stepglm[backward]+LDA 0.822 0.742 0.782
Lasso+XGBoost 0.859 0.703 0.781
Stepglm[both]+XGBoost 0.859 0.703 0.781
Stepglm[both] 0.821 0.737 0.779
Stepglm[backward] 0.821 0.737 0.779
RF+plsRglm 0.787 0.770 0.778
plsRglm 0.779 0.775 0.777
Lasso+NaiveBayes 0.778 0.771 0.775
Stepglm[both]+NaiveBayes 0.780 0.768 0.774
Stepglm[backward]+NaiveBayes 0.780 0.768 0.774
glmBoost+NaiveBayes 0.771 0.771 0.771
NaiveBayes 0.760 0.753 0.756
RF+NaiveBayes 0.761 0.741 0.751
SVM 0.803 0.687 0.745
glmBoost+SVM 0.812 0.655 0.734
XGBoost 0.787 0.675 0.731
Stepglm[backward]+SVM 0.842 0.619 0.731
RF+SVM 0.777 0.667 0.722
Lasso+SVM 0.780 0.653 0.717
Stepglm[both]+SVM 0.829 0.600 0.715
0
0.5
1
AUC
1
0.9
0.8
0.7
0.6
Cohort
Derivation cohort
Validation cohort
