# Supplementary figures and images for: Development and internal validation of an interpretable machine learning model to predict coagulopathy following extracorporeal membrane oxygenation: a retrospective multicenter study
Source: Scand J Trauma Resusc Emerg Med. 2026 Jan 28;34:45. doi: 10.1186/s13049-026-01564-x (PMC12924354; doi:10.1186/s13049-026-01564-x)

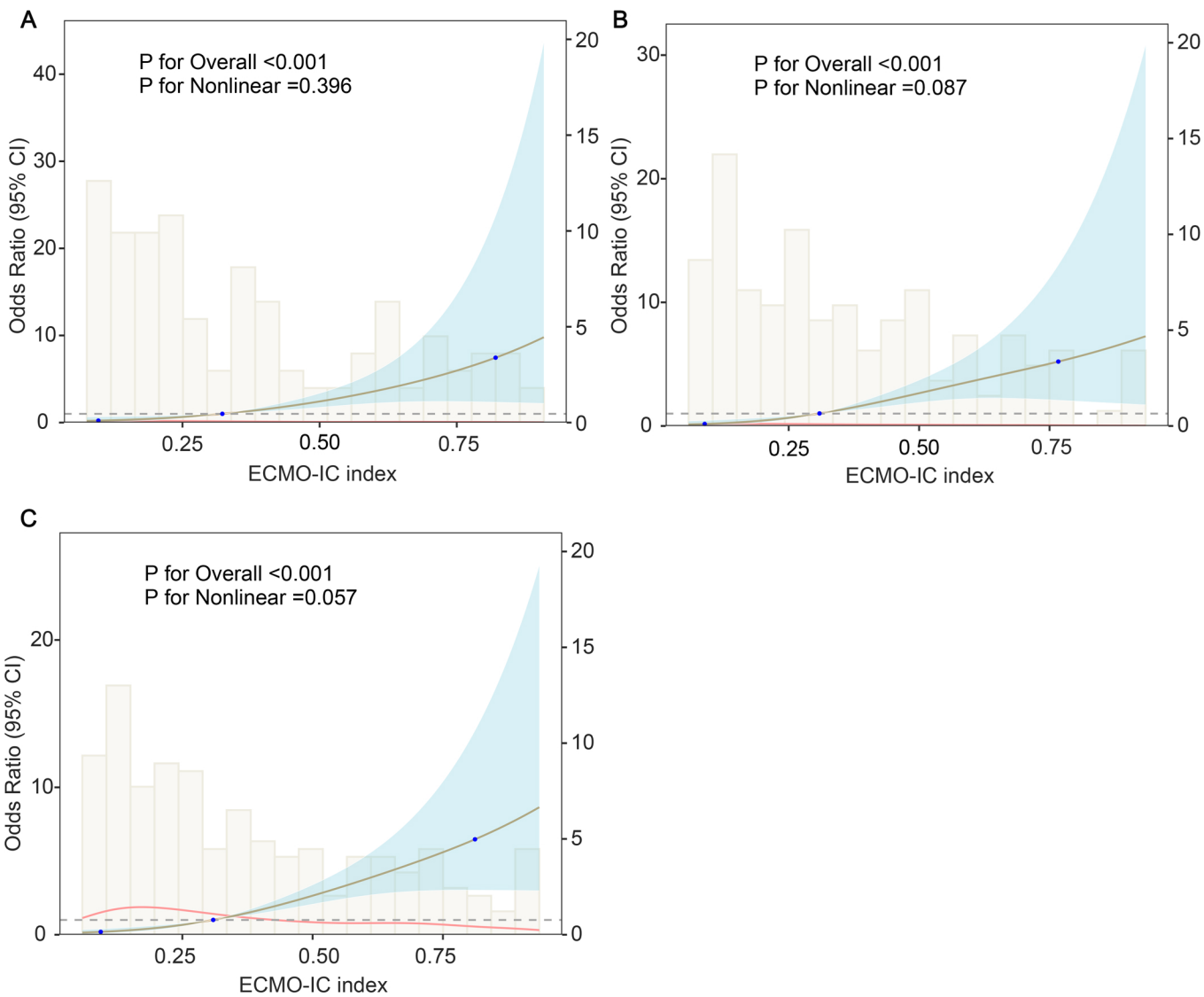

Supplement: Supplementary file 2 — Supplementary Material 2. Figure S2. Potential nonlinear for the levels of ECMO-IC index with ECMO-IC risk measured by restricted cubic spline regression with optimal knots. (A) Derivation cohort; (B)Validation cohort; (C) Entire cohort. The brown line and shadow area represent the estimated OR and the 95% CI. [file 13049_2026_1564_MOESM2_ESM.pdf]

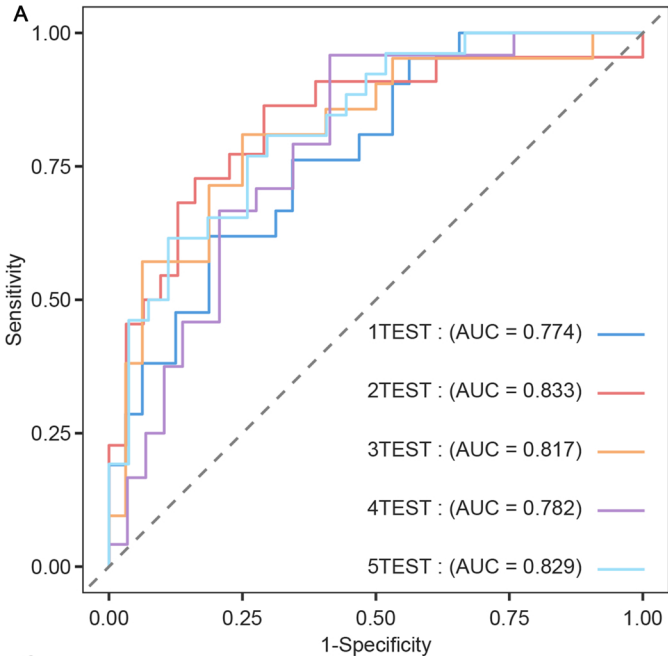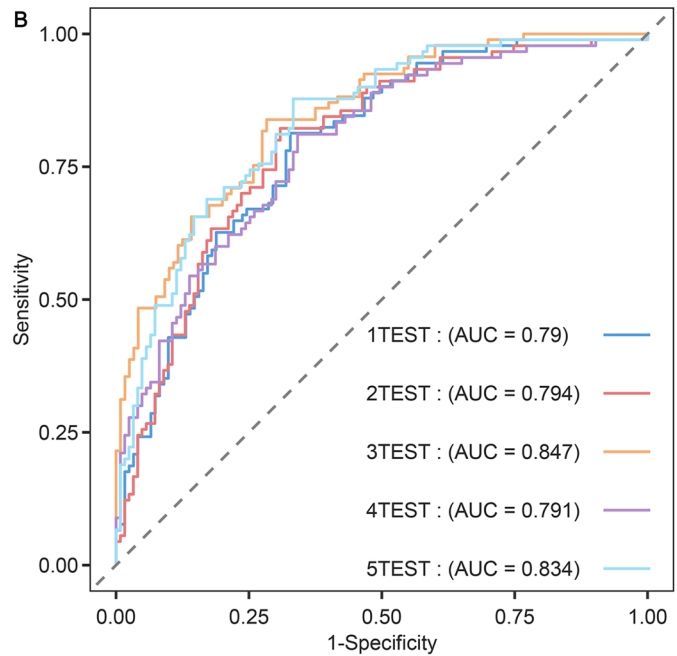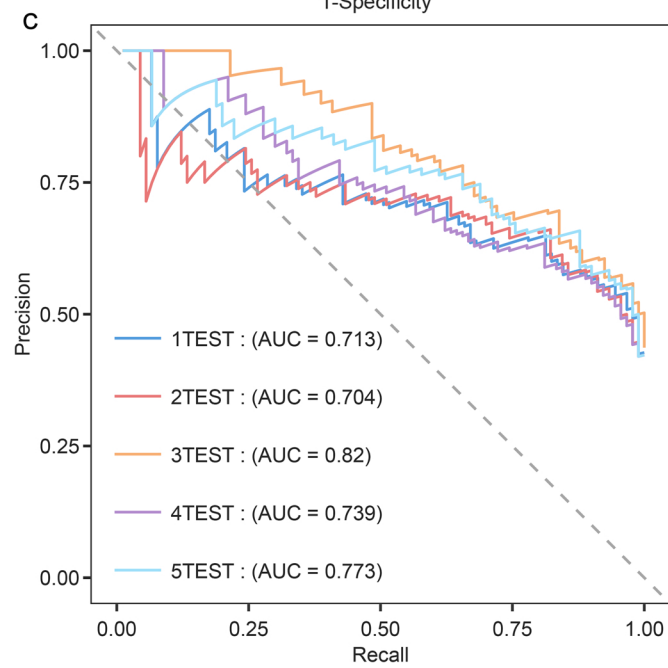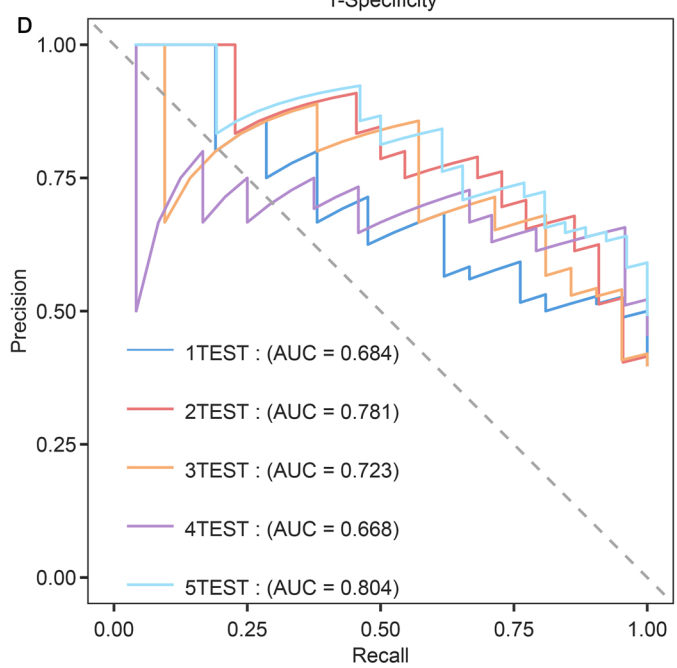

Supplement: Supplementary file 3 — Supplementary Material 3. FigureS3. 5-fold and Bootstrap cross-validation applied to evaluate the reliability and stability ability of ECMO-IC index. (A, B) ROC analysis through 5-fold (A) and Bootstrap (B) cross-validation. (C, D) PR analysis through 5-fold (C) and Bootstrap (D) cross-validation. [file 13049_2026_1564_MOESM3_ESM.pdf]

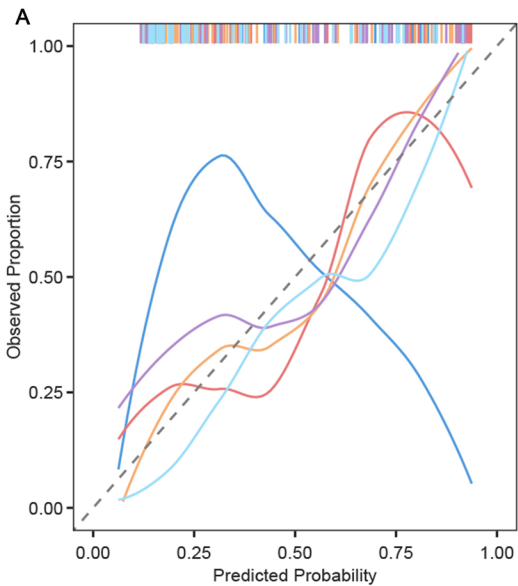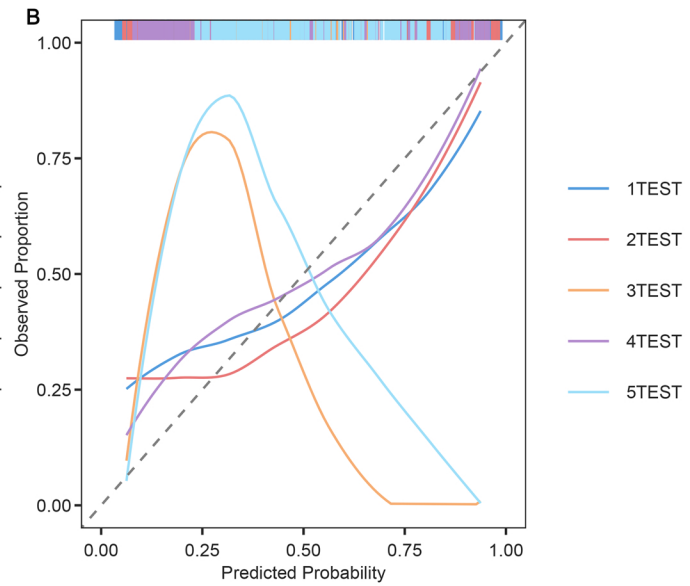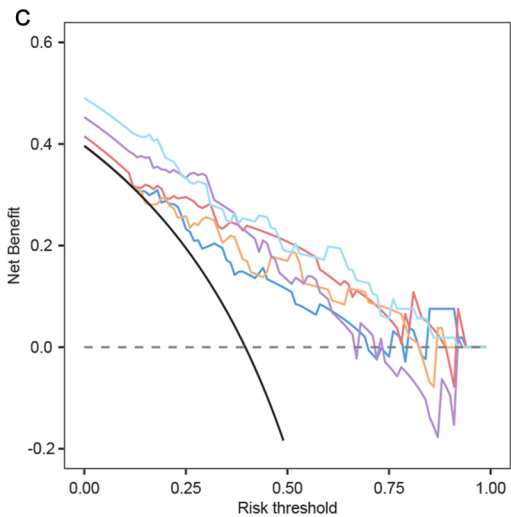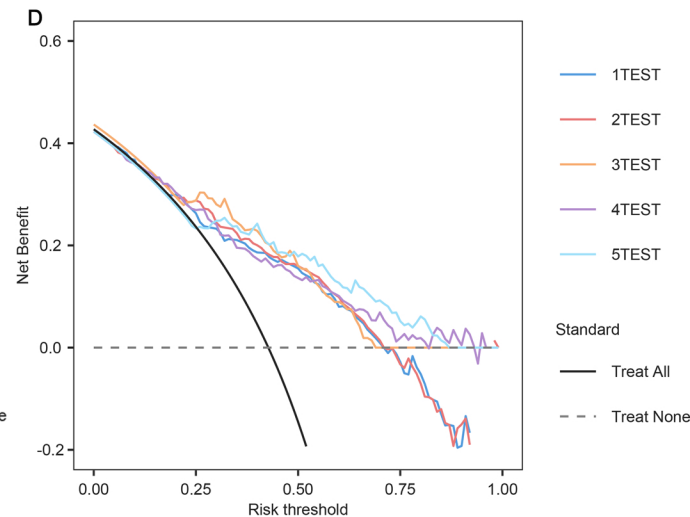

Supplement: Supplementary file 4 — Supplementary Material 4. FigureS4. 5-fold and Bootstrap cross-validation applied to evaluate the reliability and stability ability of ECMO-IC index. (A, B) Calibration curves analysis through 5-fold (A) and Bootstrap (B) cross-validation. (C, D) DCA analysis through 5-fold (C) and Bootstrap (D) cross-validation. [file 13049_2026_1564_MOESM4_ESM.pdf]
