# Supplementary material for: Development and internal validation of an interpretable machine learning model to predict coagulopathy following extracorporeal membrane oxygenation: a retrospective multicenter study
Source: Scand J Trauma Resusc Emerg Med. 2026 Jan 28;34:45. doi: 10.1186/s13049-026-01564-x (PMC12924354; doi:10.1186/s13049-026-01564-x)
Supplement: Supplementary file 5 — Supplementary Material 5. [file 13049_2026_1564_MOESM5_ESM.pdf]

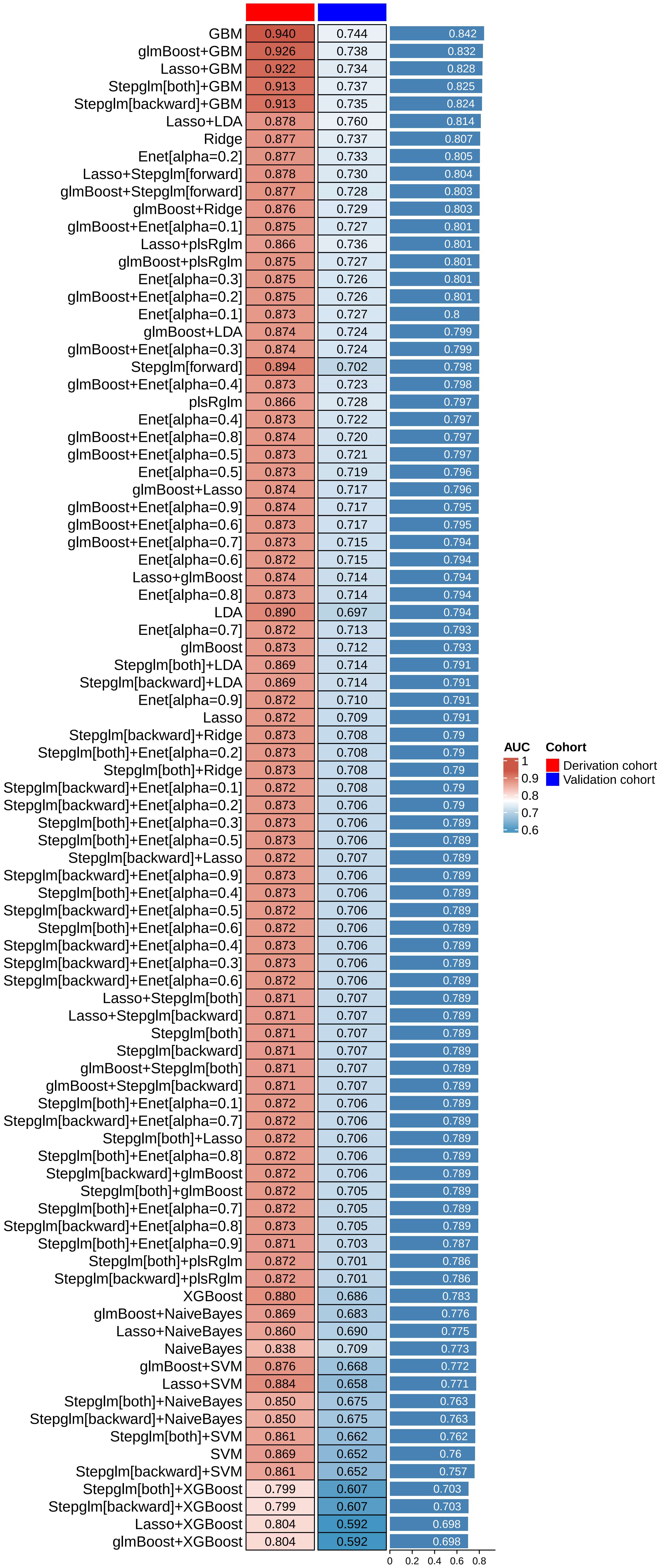

GBM 0.940 0.744 0.842
glmBoost+GBM 0.926 0.738 0.832
Lasso+GBM 0.922 0.734 0.828
Stepglm[both]+GBM 0.913 0.737 0.825
Stepglm[backward]+GBM 0.913 0.735 0.824
Lasso+LDA 0.878 0.760 0.814
Ridge 0.877 0.737 0.807
Enet[alpha=0.2] 0.877 0.733 0.805
Lasso+Stepglm[forward] 0.878 0.730 0.804
glmBoost+Stepglm[forward] 0.877 0.728 0.803
glmBoost+Ridge 0.876 0.729 0.803
glmBoost+Enet[alpha=0.1] 0.875 0.727 0.801
Lasso+plsRglm 0.866 0.736 0.801
glmBoost+plsRglm 0.875 0.727 0.801
Enet[alpha=0.3] 0.875 0.726 0.801
glmBoost+Enet[alpha=0.2] 0.875 0.726 0.801
Enet[alpha=0.1] 0.873 0.727 0.8
glmBoost+LDA 0.874 0.724 0.799
glmBoost+Enet[alpha=0.3] 0.874 0.724 0.799
Stepglm[forward] 0.894 0.702 0.798
glmBoost+Enet[alpha=0.4] 0.873 0.723 0.798
plsRglm 0.866 0.728 0.797
Enet[alpha=0.4] 0.873 0.722 0.797
glmBoost+Enet[alpha=0.8] 0.874 0.720 0.797
glmBoost+Enet[alpha=0.5] 0.873 0.721 0.797
Enet[alpha=0.5] 0.873 0.719 0.796
glmBoost+Lasso 0.874 0.717 0.796
glmBoost+Enet[alpha=0.9] 0.874 0.717 0.795
glmBoost+Enet[alpha=0.6] 0.873 0.717 0.795
glmBoost+Enet[alpha=0.7] 0.873 0.715 0.794
Enet[alpha=0.6] 0.872 0.715 0.794
Lasso+glmBoost 0.874 0.714 0.794
Enet[alpha=0.8] 0.873 0.714 0.794
LDA 0.890 0.697 0.794
Enet[alpha=0.7] 0.872 0.713 0.793
glmBoost 0.873 0.712 0.793
Stepglm[both]+LDA 0.869 0.714 0.791
Stepglm[backward]+LDA 0.869 0.714 0.791
Enet[alpha=0.9] 0.872 0.710 0.791
Lasso 0.872 0.709 0.791
Stepglm[backward]+Ridge 0.873 0.708 0.79
Stepglm[both]+Enet[alpha=0.2] 0.873 0.708 0.79
Stepglm[both]+Ridge 0.873 0.708 0.79
Stepglm[backward]+Enet[alpha=0.1] 0.872 0.708 0.79
Stepglm[backward]+Enet[alpha=0.2] 0.873 0.706 0.79
Stepglm[both]+Enet[alpha=0.3] 0.873 0.706 0.789
Stepglm[both]+Enet[alpha=0.5] 0.873 0.706 0.789
Stepglm[backward]+Lasso 0.872 0.707 0.789
Stepglm[backward]+Enet[alpha=0.9] 0.873 0.706 0.789
Stepglm[both]+Enet[alpha=0.4] 0.873 0.706 0.789
Stepglm[backward]+Enet[alpha=0.5] 0.872 0.706 0.789
Stepglm[both]+Enet[alpha=0.6] 0.872 0.706 0.789
Stepglm[backward]+Enet[alpha=0.4] 0.873 0.706 0.789
Stepglm[backward]+Enet[alpha=0.3] 0.873 0.706 0.789
Stepglm[backward]+Enet[alpha=0.6] 0.872 0.706 0.789
Lasso+Stepglm[both] 0.871 0.707 0.789
Lasso+Stepglm[backward] 0.871 0.707 0.789
Stepglm[both] 0.871 0.707 0.789
Stepglm[backward] 0.871 0.707 0.789
glmBoost+Stepglm[both] 0.871 0.707 0.789
glmBoost+Stepglm[backward] 0.871 0.707 0.789
Stepglm[both]+Enet[alpha=0.1] 0.872 0.706 0.789
Stepglm[backward]+Enet[alpha=0.7] 0.872 0.706 0.789
Stepglm[both]+Lasso 0.872 0.706 0.789
Stepglm[both]+Enet[alpha=0.8] 0.872 0.706 0.789
Stepglm[backward]+glmBoost 0.872 0.706 0.789
Stepglm[both]+glmBoost 0.872 0.705 0.789
Stepglm[both]+Enet[alpha=0.7] 0.872 0.705 0.789
Stepglm[backward]+Enet[alpha=0.8] 0.873 0.705 0.789
Stepglm[both]+Enet[alpha=0.9] 0.871 0.703 0.787
Stepglm[both]+plsRglm 0.872 0.701 0.786
Stepglm[backward]+plsRglm 0.872 0.701 0.786
XGBoost 0.880 0.686 0.783
glmBoost+NaiveBayes 0.869 0.683 0.776
Lasso+NaiveBayes 0.860 0.690 0.775
NaiveBayes 0.838 0.709 0.773
glmBoost+SVM 0.876 0.668 0.772
Lasso+SVM 0.884 0.658 0.771
Stepglm[both]+NaiveBayes 0.850 0.675 0.763
Stepglm[backward]+NaiveBayes 0.850 0.675 0.763
Stepglm[both]+SVM 0.861 0.662 0.762
SVM 0.869 0.652 0.76
Stepglm[backward]+SVM 0.861 0.652 0.757
Stepglm[both]+XGBoost 0.799 0.607 0.703
Stepglm[backward]+XGBoost 0.799 0.607 0.703
Lasso+XGBoost 0.804 0.592 0.698
glmBoost+XGBoost 0.804 0.592 0.698
0 0.2 0.4 0.6 0.8
AUC
1
0.9
0.8
0.7
0.6
Cohort
Derivation cohort
Validation cohort
