# Supplementary material for: Development and internal validation of an interpretable machine learning model to predict coagulopathy following extracorporeal membrane oxygenation: a retrospective multicenter study
Source: Scand J Trauma Resusc Emerg Med. 2026 Jan 28;34:45. doi: 10.1186/s13049-026-01564-x (PMC12924354; doi:10.1186/s13049-026-01564-x)
Supplement: Supplementary file 6 — Supplementary Material 6. [file 13049_2026_1564_MOESM6_ESM.pdf]

Bullet Edits

EDITORIAL CERTIFICATE

This document certifies that the paper listed below was edited and proofread for proper English language, grammar, punctuation, spelling, and overall style by one or more than one highly qualified native speakers at Bullet Edits. All of the suggested amendments were tracked with the Microsoft Word "Track Changes" feature. Therefore, the author had the option to reject or accept each change individually.

Manuscript Title:

Interpretable machine learning model to predict coagulopathy following extracorporeal membrane oxygenation: A multicenter cohort study

Anti-counterfeiting Code:

12eb97e901f36001c976b3997f746012

Date Issued:

2025-10-28 08:29:35

Bullet Edits is a registered company headquartered in the UK with a global presence. We offer a range of editing, proofreading services to authors. Our Ph.D. editors are all native English speakers from the USA and UK. Authors who work with Bullet Edits are guaranteed excellent language quality and timely delivery.

Address: BULLET EDITS LIMITED, 85 Great Portland Street, London,UK
Tel: UK. (+44)20457 70286 / US.(+1)312-313-9179
Web: www.bulletedits.cn/Email: info@bulletedits.cn
VAT: GB 378 9316 43

BULLET EDITS LIMITED ★ COMPANY NO. 13345261 SEAL ENGLAND AND WALES ★
