# Supplementary material for: Development and internal validation of an interpretable machine learning model to predict coagulopathy following extracorporeal membrane oxygenation: a retrospective multicenter study
Source: Scand J Trauma Resusc Emerg Med. 2026 Jan 28;34:45. doi: 10.1186/s13049-026-01564-x (PMC12924354; doi:10.1186/s13049-026-01564-x)
Supplement: Supplementary file 18 — Supplementary Material 18. [file 13049_2026_1564_MOESM18_ESM.docx]

数据整理

setwd("D:\\科研论文\\ECMO研究\\临床研究\\ECMO 凝血病\\数据分析\\模型评估")#设置工作目录

inputfile1="RS.txt" #生存时间数据

inputfile2="DT.txt" #差异基因表达数据

time_data<-read.table(inputfile1,header = T,sep = "\t",check.names = F)

geneEXP<-read.table(inputfile2,header = T,sep ="\t",check.names = F)

head(time_data)

head(geneEXP)

merger_data<-merge(time_data,geneEXP,by="ID")

write.table(merger_data,"DTRS.txt",sep = "\t",row.names = F,quote = F)

ROC曲线

setwd("D:\\科研论文\\ECMO研究\\临床研究\\ECMO 凝血病\\数据分析\\模型评估")#设置工作目录

data=read.table("DTRS.txt",sep="\t",header=T,check.names=F,row.names = 1)

library(pROC)

gmodelA <- roc(DIC~RS, data = data,smooth=F)

plot(gmodelA, print.auc=TRUE, print.thres=F, main = "ROC CURVE", col= "#6E568C",print.thres.col="#6E568C",identity.col="#6E568C",

identity.lty=1,identity.lwd=1)

pdf(file="DCA.pdf",width=4.5, height=4.5)

bioCol=c("#0066FF","#FF0000","#6E568C","#7CC767","#223D6C","#D20A13","#FFD121","#088247","#11AA4D")

DCA

setwd("D:\\科研论文\\ECMO研究\\临床研究\\ECMO 凝血病\\数据分析\\模型评估")#设置工作目录

source("dca.R")

library(nricens)

library(rms)

library(foreign)

dev=read.table("DTRS.txt",sep="\t",header=T,check.names=F,row.names = 1)

modelA <- glm(DIC~RS, data = dev, family = binomial(link="logit"),x=TRUE)

summary(modelA)

dev$ECMOIC_index<- predict(newdata=dev,modelA,"response")

#Decision Curve Analysis

pdf(file="DCA.pdf",width=4.5, height=4.5)

dca(data=dev, outcome="DIC", predictors=c("ECMOIC_index"),smooth="TRUE", probability=c("TRUE"))

dev.off()

模型比较

数据处理

setwd("D:\\科研论文\\ECMO研究\\临床研究\\ECMO 凝血病\\数据分析\\模型比较")#设置工作目录

inputfile1="RS.txt" #生存时间数据

inputfile2="ET.txt" #差异基因表达数据

time_data<-read.table(inputfile1,header = T,sep = "\t",check.names = F)

geneEXP<-read.table(inputfile2,header = T,sep ="\t",check.names = F)

head(time_data)

head(geneEXP)

merger_data<-merge(time_data,geneEXP,by="ID")

write.table(merger_data,"mROC.txt",sep = "\t",row.names = F,quote = F)

mROC

library(pROC)

setwd("D:\\科研论文\\ECMO研究\\临床研究\\ECMO 凝血病\\数据分析\\模型比较")#设置工作目录

data=read.table("mROC.txt",sep="\t",header=T,check.names=F,row.names = 1)

roc1<-roc(data$DIC, data$RS)

roc2<-roc(data$DIC, data$APACHEII)

roc3<-roc(data$DIC, data$SOFA)

pdf(file="mROC.pdf",width=5,height=5)

plot(roc1,col="#FF0000",legacy.axes=T)

plot(roc2,add=TRUE, col="#FF9900")

plot(roc3,add=TRUE, col="#0066FF")

round(auc(roc1),3)##AUC

round(ci(roc1),3)##95%CI

round(auc(roc2),3)##AUC

round(ci(roc2),3)##95%CI

round(auc(roc3),3)##AUC

round(ci(roc3),3)##95%CI

legend("bottomright",legend=c("ECMO IC index AUC=0.811",

"APACHEII AUC=0.676", "SOFA AUC=0.610"), col=c("#FF0000","#FF9900","#0066FF"),lty=1, lwd = 2)

dev.off()

DCA

setwd("D:\\科研论文\\ECMO研究\\临床研究\\ECMO 凝血病\\数据分析\\模型比较")#设置工作目录

source("dca.R")

library(nricens)

library(rms)

library(foreign)

dev=read.table("mROC.txt",sep="\t",header=T,check.names=F,row.names = 1)

modelA <- glm(DIC~RS, data = dev, family = binomial(link="logit"),x=TRUE)

summary(modelA)

dev$ECMOIC_index<- predict(newdata=dev,modelA,"response")

modelB <- glm(DIC ~APACHEII, data = dev, family = binomial(link="logit"),x=TRUE)

summary(modelB)

dev$APACHEII<- predict(newdata=dev,modelB,"response")

modelC <- glm(DIC ~SOFA, data = dev, family = binomial(link="logit"),x=TRUE)

summary(modelC)

dev$SOFA<- predict(newdata=dev,modelC,"response")

#Decision Curve Analysis

pdf(file="DCA.pdf", width=5.5, height=5)

dca(data=dev, outcome="DIC", predictors=c("ECMOIC_index", "APACHEII","SOFA"),smooth="TRUE", probability=c("TRUE", "TRUE","TRUE"))

dev.off()

中介分析

library(mediation)

setwd("D:\\科研论文\\ECMO研究\\临床研究\\ECMO 凝血病\\数据分析\\模型中介")

date<-read.table("ETRS.txt", header = T,sep = "\t",check.names = F)

set.seed(12345)

Y1<-glm(Outcome~RSR,data=date,family=binomial("probit"))#表示模型1即X对Y的总效应，Y是二分类变量，所以使用glm函数

summary(Y1)#查看Y1结果

Y2<-glm(DIC~RSR,data=date, family=binomial("probit"))#模型2，M是二分类变量，所以使用glm函数

summary(Y2)

Y3<-glm(Outcome~DIC+RSR,data=date,family=binomial("probit"))#模型3

summary(Y3)

med<-mediate(Y2,Y3,treat='RSR',mediator = 'DIC',boot =T,sims = 1000)

summary(med)

plot(med)

Y2<-lm(M~X,data=date)#模型2，M是连续性变量，所以使用lm函数

summary(Y2)

SHAP

library(shapviz)

library(xgboost)

library(pROC)

require(Matrix)

require(data.table)

setwd("D:\\科研论文\\ECMO研究\\临床研究\\ECMO 凝血病\\数据分析\\模型解释")#设置工作目录

df=read.table("ET.txt",sep="\t",header=T,check.names=F, row.names = 1)

head(df)

#把结局变量生存状态转换为因子型

df$Status<-factor(df$Status,levels = c(0,1),labels = c("alive","dead"))

#查看数据结构

str(df)

#将数据转化为稀疏矩阵

sparse_matrix <- sparse.model.matrix(Status~ ., data = df)[,-1]

#查看前几行

head(sparse_matrix)

output_vector = df[,1] == "dead"

#建立模型！

bst <- xgboost(data = sparse_matrix, label = output_vector, max_depth = 4,

eta = 1, nthread = 2, nrounds =10,objective = "binary:logistic")

shap <- shapviz(bst,X_pred =data.matrix(df[,-1]))

sv_waterfall(shap,

row_id = 3,

max_display = 30,

fill_colors=c("#FF0000", "#0085FF"))

sv_force(shap,

row_id = 2,

max_display = 30,

fill_colors=c("#FF0000", "#0085FF"))

sv_importance(shap, kind = "beeswarm",

max_display = 30)

# 绘制条形图

sv_importance(shap,fill = "#0085FF",

max_display = 30)

sv_dependence(shap,

v = "", ##这个地方注意要换成自己的基因哦！！

color_var = NULL)

sv_dependence(shp,

v = c("nodes",

"pgr",

"hormon",

"age"))

#计算并可视化SHAP值的交互作用，使用sv_interaction函数。

shp_i <- shapviz(bst,

X_pred =data.matrix(df[,-1]),

interactions = TRUE)

sv_interaction(shp_i)+

theme(axis.text.x = element_text(angle = 45, vjust = 1, hjust = 1))

setwd("D:\\科研论文\\科研学术\\HNC临床研究\\PTC临床研究\\SHAP")#设置工作目录

inputfile1="Feature.txt" #生存时间数据

inputfile2="Training cohort.txt" #差异基因表达数据

time_data<-read.table(inputfile1,header = T,sep = "\t",check.names = F)

geneEXP<-read.table(inputfile2,header = T,sep = "\t",check.names = F)

head(time_data)

head(geneEXP)

merger_data<-merge(time_data,geneEXP,by="id")

write.table(merger_data,"XGBoost.txt",sep = "\t",row.names = F,quote = F)

setwd("D:\\科研论文\\科研学术\\HNC临床研究\\PTC临床研究\\SHAP")#设置工作目录

inputfile1="Feature.txt" #生存时间数据

inputfile2="Internal validation cohort.txt" #差异基因表达数据

time_data<-read.table(inputfile1,header = T,sep = "\t",check.names = F)

geneEXP<-read.table(inputfile2,header = T,sep = "\t",check.names = F)

head(time_data)

head(geneEXP)

merger_data<-merge(time_data,geneEXP,by="id")

write.table(merger_data,"XGBoost.txt",sep = "\t",row.names = F,quote = F)

library(shapviz)

library(xgboost)

library(pROC)

require(Matrix)

require(data.table)

setwd("D:\\科研论文\\科研学术\\HNC临床研究\\PTC临床研究\\SHAP")#设置工作目录

df=read.table("XGBoost.txt",sep="\t",header=T,check.names=F, row.names = 1)

head(df)

#把结局变量生存状态转换为因子型

df$Status<-factor(df$Status,levels = c(0,1),labels = c("alive","dead"))

#查看数据结构

str(df)

#将数据转化为稀疏矩阵

sparse_matrix <- sparse.model.matrix(Status~ ., data = df)[,-1]

#查看前几行

head(sparse_matrix)

output_vector = df[,1] == "dead"

#建立模型！

bst <- xgboost(data = sparse_matrix, label = output_vector, max_depth = 4,

eta = 1, nthread = 2, nrounds =10,objective = "binary:logistic")

shap <- shapviz(bst,X_pred =data.matrix(df[,-1]))

sv_waterfall(shap,

row_id = 3,

max_display = 30,

fill_colors=c("#FF0000", "#0085FF"))

sv_force(shap,

row_id = 2,

max_display = 30,

fill_colors=c("#FF0000", "#0085FF"))

sv_importance(shap, kind = "beeswarm",

max_display = 30)

# 绘制条形图

sv_importance(shap,fill = "#0085FF",

max_display = 30)

sv_dependence(shap,

v = "", ##这个地方注意要换成自己的基因哦！！

color_var = NULL)

sv_dependence(shp,

v = c("nodes",

"pgr",

"hormon",

"age"))

#计算并可视化SHAP值的交互作用，使用sv_interaction函数。

shp_i <- shapviz(bst,

X_pred =data.matrix(df[,-1]),

interactions = TRUE)

sv_interaction(shp_i)+

theme(axis.text.x = element_text(angle = 45, vjust = 1, hjust = 1))

LASSO回归分析

library(glmnet)

set.seed(123)

setwd("D:\\科研论文\\ECMO研究\\临床研究\\ECMO 凝血病\\数据分析\\模型LASSO")

#设置工作目录

data<-read.table("DT.txt",header=T,sep="\t",row.names = 1,check.names = F,stringsAsFactors = F)

v1<-as.matrix(data[,c(2:ncol(data))])

v2 <- as.matrix(data$DIC)

fit <- glmnet(v1, v2, alpha=1,family='binomial')

plot(fit, xvar = "lambda", label =FALSE)

cvfit <- cv.glmnet(v1, v2, alpha=1)

plot(cvfit)

abline(v=log(c(cvfit$lambda.min,cvfit$lambda.1se)),lty="dashed")

cvfit$lambda.min

library(ggplot2)

library(ggsci)

x <- coef(fit)

tmp <- as.data.frame(as.matrix(x))

tmp$coef <- row.names(tmp)

tmp <- reshape::melt(tmp, id = "coef")

tmp$variable <- as.numeric(gsub("s", "", tmp$variable))

tmp$coef <- gsub('_','-',tmp$coef)

tmp$lambda <- fit$lambda[tmp$variable+1] # extract the lambda values

tmp$norm <- apply(abs(x[-1,]),2,sum)[tmp$variable+1]#compute L1 norm

head(tmp)

write.table(tmp,"tmp.txt",sep = "\t",row.names = F,quote = F)

head(tmp)

write.table(tmp,"TMP.txt",sep = "\t",row.names = F,quote = F)

tmp<-read.table("TMP.txt",header=T,sep="\t",check.names = F,stringsAsFactors = F)

ggplot(tmp,aes(log(lambda),value,color = coef)) +

geom_vline(xintercept = log(cvfit$lambda.min),size=0.8,color='grey60',alpha=0.8,linetype=2)+

geom_line(size=1) +

xlab("Lambda (log scale)") +

#xlab("L1 norm")+

ylab('Coefficients')+

theme_bw(base_rect_size = 1)+

scale_color_manual(values = c(pal_npg()(10),pal_d3()(10),pal_jco()(7)))+

scale_x_continuous(expand = c(0.01,0.01))+

scale_y_continuous(expand = c(0.01,0.01))+

theme(panel.grid = element_blank(),

axis.title = element_text(size=13,color='black'),

axis.text = element_text(size=13,color='black'),

legend.title = element_blank(),

legend.text = element_text(size=13,color='black'),

legend.position = 'right')+

#annotate('text',x = -3.3,y=0.26,label='Optimal Lambda = 0.012',color='black')+

guides(col=guide_legend(ncol = 1))

tmp$coef2 <- ifelse(tmp$norm==max(tmp$norm),tmp$coef,NA)

ggplot(tmp,aes(norm,value,color = coef)) +

geom_vline(xintercept = exp(cvfit$lambda.min),size=0.8,color='grey60',alpha=0.8,linetype=2)+

geom_line(size=1) +

xlab("L1 norm") +

ylab('Coefficients')+

theme_bw(base_rect_size = 1)+

scale_color_manual(values = c(pal_npg()(10),pal_d3()(10),pal_jco()(7)))+

scale_x_continuous(expand = c(0.01,0.01))+

scale_y_continuous(expand = c(0.01,0.01))+

theme(axis.title = element_text(size=13,color='black'),

axis.text = element_text(size=13,color='black'),

legend.title = element_blank(),

legend.text = element_text(size=13,color='black'), legend.position = 'none')+

ggrepel::geom_text_repel(aes(2.7,value,label=coef2),fontface="bold")

ROC曲线

setwd("F:\\科研论文\\科研学术\\HNC临床研究\\PTC临床研究\\数据分析\\模型评估")#设置工作目录

inputfile1="id.txt" #生存时间数据

inputfile2="PTC.txt" #差异基因表达数据

time_data<-read.table(inputfile1,header = T,sep = "\t",check.names = F)

geneEXP<-read.table(inputfile2,header = T,sep = "\t",check.names = F)

head(time_data)

head(geneEXP)

merger_data<-merge(time_data,geneEXP,by="id")

write.table(merger_data,"PTCROC.txt",sep = "\t",row.names = F,quote = F)

data=read.table("PTCROC.txt",sep="\t",header=T,check.names=F,row.names = 1)

library(pROC)

gmodelA <- roc(Delphian_LN~RS, data = data,smooth=F)

plot(gmodelA, print.auc=TRUE, print.thres=F, main = "ROC CURVE", col= "#6E568C",print.thres.col="#6E568C",identity.col="#6E568C",

identity.lty=1,identity.lwd=1)

bioCol=c("#0066FF","#FF0000","#6E568C","#7CC767","#223D6C","#D20A13","#FFD121","#088247","#11AA4D")

setwd("F:\\科研论文\\科研学术\\HNC临床研究\\PTC临床研究\\数据分析\\模型评估")#设置工作目录

source("dca.R")

library(nricens)

library(rms)

library(foreign)

dev=read.table("PTCROC.txt",sep="\t",header=T,check.names=F,row.names = 1)

modelA <- glm(Delphian_LN~RS, data = dev, family = binomial(link="logit"),x=TRUE)

summary(modelA)

dev$Treg_score<- predict(newdata=dev,modelA,"response")

#Decision Curve Analysis

pdf(file="DCA.pdf",width=5, height=5.5)

dca(data=dev, outcome="Status", predictors=c("Treg_score"),smooth="TRUE", probability=c("TRUE"))

dev.off()

模型的比较

library(rms)

library(foreign)

setwd("F:\\科研论文\\科研学术\\HNC临床研究\\PTC临床研究\\数据分析\\模型比较")

data=read.table("PTC.txt",sep="\t",header=T,check.names=F,row.names = 1)

ddist <- datadist(data)

options(datadist='ddist')

modelA <- glm(Delphian_LN~ Sex + Age + B_ultrasound_tumor_size, data = data, family = binomial(link="logit"))

summary(modelA)

cbind(coef= coef(modelA),confint(modelA))

exp(cbind(OR= coef(modelA),confint(modelA)))

RS1<- predict(newdata=data,modelA,"response")

write.table(cbind(id=rownames(cbind(data[,1:30],RS1)),cbind(data[,1:30],RS1)),"RiskScore.txt",sep="\t",quote=F,row.names=F)

data=read.table("RiskScore.txt",sep="\t",header=T,check.names=F,row.names = 1)

ddist <- datadist(data)

options(datadist='ddist')

modelA <- glm(Delphian_LN~ B_ultrasound_left_CLN + B_ultrasound_left_3_region_LN + B_ultrasound_right_CLN +B_ultrasound_right_3_region_LN + CT_CLN, data = data, family = binomial(link="logit"))

summary(modelA)

cbind(coef= coef(modelA),confint(modelA))

exp(cbind(OR= coef(modelA),confint(modelA)))

RS2<- predict(newdata=data,modelA,"response")

write.table(cbind(id=rownames(cbind(data[,1:31],RS2)),cbind(data[,1:32],RS2)),"RiskScore.txt",sep="\t",quote=F,row.names=F)

mROC

library(pROC)

setwd("F:\\科研论文\\科研学术\\HNC临床研究\\PTC临床研究\\数据分析\\模型比较")

data=read.table("RiskScore.txt",sep="\t",header=T,check.names=F,row.names = 1)

roc1<-roc(data$Delphian_LN, data$RS)

roc2<-roc(data$Delphian_LN, data$RS1)

roc3<-roc(data$Delphian_LN, data$RS2)

pdf(file="mROC.pdf",width=5,height=5)

plot(roc1,col="#FF0000",legacy.axes=T)

plot(roc2,add=TRUE, col="#FF9900")

plot(roc3,add=TRUE, col="#0066FF")

round(auc(roc1),3)##AUC

round(ci(roc1),3)##95%CI

round(auc(roc2),3)##AUC

round(ci(roc2),3)##95%CI

round(auc(roc3),3)##AUC

round(ci(roc3),3)##95%CI

legend("bottomright",legend=c("DLNM index AUC=0.763",

"Li model AUC=0.649", "Zhou model AUC=0.656"), col=c("#FF0000","#FF9900","#0066FF"),lty=1, lwd = 2)

dev.off()

DCA

setwd("F:\\科研论文\\科研学术\\HNC临床研究\\PTC临床研究\\数据分析\\模型比较")

source("dca.R")

library(nricens)

library(rms)

library(foreign)

dev=read.table("DCA.txt",sep="\t",header=T,check.names=F,row.names = 1)

modelA <- glm(Delphian_LN~RS, data = dev, family = binomial(link="logit"),x=TRUE)

summary(modelA)

dev$DLNM_index<- predict(newdata=dev,modelA,"response")

modelB <- glm(Delphian_LN ~RS1, data = dev, family = binomial(link="logit"),x=TRUE)

summary(modelB)

dev$Li_model<- predict(newdata=dev,modelB,"response")

modelC <- glm(Delphian_LN ~RS2, data = dev, family = binomial(link="logit"),x=TRUE)

summary(modelC)

dev$Zhou_model<- predict(newdata=dev,modelC,"response")

#Decision Curve Analysis

pdf(file="DCA.pdf", width=5.5, height=5)

dca(data=dev, outcome="Delphian_LN", predictors=c("DLNM_index", "Li_model","Zhou_model"),smooth="TRUE", probability=c("TRUE", "TRUE","TRUE"))

dev.off()

SHAP

library(shapviz)

library(xgboost)

library(pROC)

require(Matrix)

require(data.table)

setwd("F:\\科研论文\\科研学术\\HNC临床研究\\PTC临床研究\\数据分析\\SHAP")#设置工作目录

df=read.table("XGBoost.txt",sep="\t",header=T,check.names=F, row.names = 1)

head(df)

#把结局变量生存状态转换为因子型

df$Status<-factor(df$Status,levels = c(0,1),labels = c("alive","dead"))

#查看数据结构

str(df)

#将数据转化为稀疏矩阵

sparse_matrix <- sparse.model.matrix(Status~ ., data = df)[,-1]

#查看前几行

head(sparse_matrix)

output_vector = df[,1] == "dead"

#建立模型！

bst <- xgboost(data = sparse_matrix, label = output_vector, max_depth = 4,

eta = 1, nthread = 2, nrounds =10,objective = "binary:logistic")

shap <- shapviz(bst,X_pred =data.matrix(df[,-1]))

sv_waterfall(shap,

row_id = 3,

max_display = 30,

fill_colors=c("#FF0000", "#0085FF"))

sv_force(shap,

row_id = 2,

max_display = 30,

fill_colors=c("#FF0000", "#0085FF"))

sv_importance(shap, kind = "beeswarm",

max_display = 30)

# 绘制条形图

sv_importance(shap,fill = "#0085FF",

max_display = 30)

sv_dependence(shap,

v = "", ##这个地方注意要换成自己的基因哦！！

color_var = NULL)

sv_dependence(shp,

v = c("nodes",

"pgr",

"hormon",

"age"))

#计算并可视化SHAP值的交互作用，使用sv_interaction函数。

shp_i <- shapviz(bst,

X_pred =data.matrix(df[,-1]),

interactions = TRUE)

sv_interaction(shp_i)+

theme(axis.text.x = element_text(angle = 45, vjust = 1, hjust = 1))

UpSetR

library(UpSetR)

library(openxlsx)

library(RColorBrewer)

setwd("D:\\科研论文\\科研学术\\MR\\ARF MR\\UpSetR")

data=read.table("UpSetR.txt", header=T, sep="\t", check.names=F)

head(data)

upset(fromList(data))

#调整与美化后的集合图#

pdf(file="UpSetR.pdf",width=12,height=7)

upset(fromList(data),

nsets=length(data),#显示数据集的所有数据,nsets = 数值调整可视化数据集数量

nintersects=30,#显示前多少个

number.angles = 0, #交互集合柱状图的柱标倾角

point.size=2, #图中点的大小

line.size=1, #图中连接线粗细

mainbar.y.label="Intersection size", #y轴的标签

main.bar.color = 'cadetblue4', #y轴柱状图颜色

matrix.color="black", #x轴点的颜色

sets.x.label="Set size", #x轴的标签

sets.bar.color=brewer.pal(3,"Set1"),#x轴柱状图的颜色;Set1中只有9个颜色，Set3中有12个颜色，Paired中有12个颜色

mb.ratio = c(0.7, 0.3), #bar plot和matrix plot图形高度的占比

order.by = "freq", #y轴矩阵排序,如"freq"频率，"degree"程度

text.scale=c(1.5,1.5,1.5,1.5,1.7,1.8), #6个参数intersection size title（y标题大小）,intersection size tick labels（y刻度标签大小）, set size title（set标题大小）, set size tick labels（set刻度标签大小）, set names（set 分类标签大小）, numbers above bars（柱数字大小）的设置

shade.color="red" #图中阴影部分的颜色

)

dev.off()

相关系数

setwd("D:\\科研论文\\ECMO研究\\临床研究\\ECMO 凝血病\\数据分析\\共线性诊断")#设置工作目录

mydata=read.table("DTCor.txt",sep="\t",header=T,check.names=F,row.names = 1)

res <- cor(mydata)

round(res, 2)

write.table(res,"DTCorR.txt",sep = "\t",row.names = F,quote = F)

模型系数

library(rms)

library(foreign)

setwd("D:\\科研论文\\ECMO研究\\临床研究\\ECMO 凝血病\\数据分析\\模型系数")

data=read.table("DT.txt",sep="\t",header=T,check.names=F,row.names = 1)

ddist <- datadist(data)

options(datadist='ddist')

modelA <- glm(DIC~ PLT+ Lac+ SII+ K+ TP+SI+RDWCV+APACHEII+ Ca, data = data, family = binomial(link="logit"))

summary(modelA)

cbind(coef= coef(modelA),confint(modelA))

exp(cbind(OR= coef(modelA),confint(modelA)))

RS1<- predict(newdata=data,modelA,"response")

write.table(cbind(id=rownames(cbind(data[,1:9],RS1)),cbind(data[,1:9],RS1)),"RS1.txt",sep="\t",quote=F,row.names=F)

library(pROC)

gmodelA <- roc(DIC~RS1, data = data,smooth=F)

plot(gmodelA, print.auc=TRUE, print.thres=F, main = "ROC CURVE", col= "#6E568C",print.thres.col="#6E568C",identity.col="#6E568C",

identity.lty=1,identity.lwd=1)

**机器模型**

# 设置工作路径

work.path <- "D:\\科研论文\\科研学术\\HNC临床研究\\PTC临床研究\\12种机器学习"; setwd(work.path)

# 设置其他路径

code.path <- file.path(work.path, "Codes")

data.path <- file.path(work.path, "InputData")

res.path <- file.path(work.path, "Results")

fig.path <- file.path(work.path, "Figures")

# 如不存在这些路径则创建路径

if (!dir.exists(data.path)) dir.create(data.path)

if (!dir.exists(res.path)) dir.create(res.path)

if (!dir.exists(fig.path)) dir.create(fig.path)

if (!dir.exists(code.path)) dir.create(code.path)

library(openxlsx)

library(seqinr)

library(plyr)

library(randomForestSRC)

library(glmnet)

library(plsRglm)

library(gbm)

library(caret)

library(mboost)

library(e1071)

library(BART)

library(MASS)

library(snowfall)

library(xgboost)

library(ComplexHeatmap)

library(RColorBrewer)

library(pROC)

# 加载模型训练以及模型评估的脚本

source(file.path(code.path, "ML.R"))

# 选择最后生成的模型类型：panML代表生成由不同算法构建的模型； multiLogistic表示抽取其他模型所用到的变量并建立多变量logistic模型

FinalModel <- c("panML", "multiLogistic")[2]

## Training Cohort ---------------------------------------------------------

# 训练集表达谱是行为基因（感兴趣的基因集），列为样本的表达矩阵（基因名与测试集保持相同类型，如同为SYMBOL或ENSEMBL等）

Train_expr <- read.table(file.path(data.path, "Training_expr.txt"), header = T, sep = "\t", row.names = 1,check.names = F,stringsAsFactors = F)

# 行为样本，列包含至少一个需要预测的二分类变量(仅支持[0，1]格式)

Train_class <- read.table(file.path(data.path, "Training_class.txt"), header = T, sep = "\t", row.names = 1,check.names = F,stringsAsFactors = F)

# 提取训练集的共有样本

comsam <- intersect(rownames(Train_class), colnames(Train_expr))

Train_expr <- Train_expr[,comsam]; Train_class <- Train_class[comsam,,drop = F]

## Validation Cohort -------------------------------------------------------

# 测试集表达谱是行为基因（感兴趣的基因集），列为样本的表达矩阵（基因名与训练集保持相同类型，如同为SYMBOL或ENSEMBL等）

Test_expr <- read.table(file.path(data.path, "Testing_expr.txt"), header = T, sep = "\t", row.names = 1,check.names = F,stringsAsFactors = F)

# 行为样本，列包含至少一个需要预测的二分类变量(仅支持[0，1]格式)，以及一列用于指定队列信息的变量

Test_class <- read.table(file.path(data.path, "Testing_class.txt"), header = T, sep = "\t", row.names = 1,check.names = F,stringsAsFactors = F)

# 提取测试集的共有样本

comsam <- intersect(rownames(Test_class), colnames(Test_expr))

Test_expr <- Test_expr[,comsam]; Test_class <- Test_class[comsam,,drop = F]

# 提取相同基因

comgene <- intersect(rownames(Train_expr),rownames(Test_expr))

Train_expr <- t(Train_expr[comgene,]) # 输入模型的表达谱行为样本，列为基因

Test_expr <- t(Test_expr[comgene,]) # 输入模型的表达谱行为样本，列为基因

# 按队列对数据分别进行标准化（根据情况调整centerFlags和scaleFlags）

## data: 需要表达谱数据（行为样本，列为基因）

## cohort：样本所属队列，为向量，不输入值时默认全表达矩阵来自同一队列

## centerFlag/scaleFlags：是否将基因均值/标准差标准化为1；

## 默认参数为NULL，表示不进行标准化；

## 为T/F时，表示对所有队列都进行/不进行标准化

## 输入由T/F组成的向量时，按顺序对队列进行处理，向量长度应与队列数一样

## 如centerFlags = c(F, F, F, T, T)，表示对第4、5个队列进行标准化，此时flag顺序应当与队列顺序一致

## 如centerFlags = c("A" = F, "C" = T, "B" = F)，表示对队列C进行标准化，此时不要求flag顺序与data一致

Train_set = scaleData(data = Train_expr, centerFlags = T, scaleFlags = T)

names(x = split(as.data.frame(Test_expr), f = Test_class$Cohort)) # 注意测试集标准化顺序与此一致

Test_set = scaleData(data = Test_expr, cohort = Test_class$Cohort, centerFlags = T, scaleFlags = T)

# summary(apply(Train_set, 2, var))

# summary(apply(Test_set, 2, var))

# lapply(split(as.data.frame(Test_set), Test_class$Cohort), function(x) summary(apply(x, 2, var))) # 测试scale结果

# Model training and validation -------------------------------------------

## method list --------------------------------------------------------

# 此处记录需要运行的模型，格式为：算法1名称[算法参数]+算法2名称[算法参数]

# 目前仅有Stepglm和Enet支持输入算法参数

methods <- read.xlsx(file.path(code.path, "methods.xlsx"), startRow = 2)

methods <- methods$Model

methods <- gsub("-| ", "", methods)

## Train the model --------------------------------------------------------

classVar = "outcome" # 设置所要预测的变量名（仅支持[0,1]二元变量格式）

min.selected.var = 5 # 设置模型最少纳入的变量数

## Pre-training 将各方法所用到的变量筛选过程汇总，以减少计算量

Variable = colnames(Train_set)

preTrain.method = strsplit(methods, "\\+") # 检视所有方法，分析各方法是否需要进行变量预筛选(pre-training)

preTrain.method = lapply(preTrain.method, function(x) rev(x)[-1]) # 删除各方法用于构建分类模型的算法，保留用于变量筛选的算法

preTrain.method = unique(unlist(preTrain.method)) # 汇总所有变量筛选算法，去除重复计算

preTrain.var <- list() # 用于保存各算法筛选的变量

set.seed(seed = 777) # 设置建模种子，使得结果可重复

for (method in preTrain.method){

preTrain.var[[method]] = RunML(method = method, # 变量筛选所需要的机器学习方法

Train_set = Train_set, # 训练集有潜在预测价值的变量

Train_label = Train_class, # 训练集分类标签

mode = "Variable", # 运行模式，Variable(筛选变量)和Model(获取模型)

classVar = classVar) # 用于训练的分类变量，必须出现在Train_class中

}

preTrain.var[["simple"]] <- colnames(Train_set)# 记录未经筛选的变量集（以便后续代码撰写），可视为使用simple方法（无筛选功能）的变量筛选结果

## Model training

model <- list() # 用于保存各模型的所有信息

set.seed(seed = 777) # 设置建模种子，使得结果可重复

Train_set_bk = Train_set # RunML有一个函数(plsRglm)无法正常传参，需要对训练集数据进行存档备份

for (method in methods){

cat(match(method, methods), ":", method, "\n")

method_name = method # 本轮算法名称

method <- strsplit(method, "\\+")[[1]] # 各步骤算法名称

if (length(method) == 1) method <- c("simple", method) # 如果本方法没有预筛选变量，则认为本方法使用simple方法进行了变量筛选

Variable = preTrain.var[[method[1]]] # 根据方法名称的第一个值，调用先前变量筛选的结果

Train_set = Train_set_bk[, Variable] # 对训练集取子集，因为有一个算法原作者写的有点问题，无法正常传参

Train_label = Train_class # 所以此处需要修改变量名称，以免函数错误调用对象

model[[method_name]] <- RunML(method = method[2], # 根据方法名称第二个值，调用构建的函数分类模型

Train_set = Train_set, # 训练集有潜在预测价值的变量

Train_label = Train_label, # 训练集分类标签

mode = "Model", # 运行模式，Variable(筛选变量)和Model(获取模型)

classVar = classVar) # 用于训练的分类变量，必须出现在Train_class中

# 如果最终模型纳入的变量数小于预先设定的下限，则认为该算法输出的结果是无意义的

if(length(ExtractVar(model[[method_name]])) <= min.selected.var) {

model[[method_name]] <- NULL

}

}

Train_set = Train_set_bk; rm(Train_set_bk) # 将数据还原，并移除备份

saveRDS(model, file.path(res.path, "model.rds")) # 报错各模型的所有中间过程

if (FinalModel == "multiLogistic"){

logisticmodel <- lapply(model, function(fit){ # 根据各算法最终获得的变量，构建多变量Logistic模型，从而以Logistic回归系数和特征表达计算单样本分类概率

tmp <- glm(formula = Train_class[[classVar]] ~ .,

family = "binomial",

data = as.data.frame(Train_set[, ExtractVar(fit)]))

tmp$subFeature <- ExtractVar(fit) # 提取当Logistic模型最终使用的预测变量

return(tmp)

})

}

saveRDS(logisticmodel, file.path(res.path, "logisticmodel.rds")) # 保存最终以多变量Logistic模型

## Evaluate the model -----------------------------------------------------

# 读取已保存的模型列表

model <- readRDS(file.path(res.path, "model.rds"))

# model <- readRDS(file.path(res.path, "logisticmodel.rds")) # 若希望使用多变量保存最终以多变量Logistic模型计算得分，请运行此行

methodsValid <- names(model)

# 根据给定表达量计算样本风险评分

# 预测概率

RS_list <- list()

for (method in methodsValid){

RS_list[[method]] <- CalPredictScore(fit = model[[method]],

new_data = rbind.data.frame(Train_set,Test_set)) # 2.0更新

}

RS_mat <- as.data.frame(t(do.call(rbind, RS_list)))

write.table(RS_mat, file.path(res.path, "RS_mat.txt"),sep = "\t", row.names = T, col.names = NA, quote = F) # 输出风险评分文件

# 根据给定表达量预测分类

Class_list <- list()

for (method in methodsValid){

Class_list[[method]] <- PredictClass(fit = model[[method]],

new_data = rbind.data.frame(Train_set,Test_set)) # 2.0更新

}

Class_mat <- as.data.frame(t(do.call(rbind, Class_list)))

#Class_mat <- cbind.data.frame(Test_class, Class_mat[rownames(Class_mat),]) # 若要合并测试集本身的样本信息文件可运行此行

write.table(Class_mat, file.path(res.path, "Class_mat.txt"), # 测试集经过算法预测出的二分类结果

sep = "\t", row.names = T, col.names = NA, quote = F)

# 提取所筛选的变量（列表格式）

fea_list <- list()

for (method in methodsValid) {

fea_list[[method]] <- ExtractVar(model[[method]])

}

# 提取所筛选的变量（数据框格式）

fea_df <- lapply(model, function(fit){

data.frame(ExtractVar(fit))

})

fea_df <- do.call(rbind, fea_df)

fea_df$algorithm <- gsub("(.+)\\.(.+$)", "\\1", rownames(fea_df))

colnames(fea_df)[1] <- "features"

write.table(fea_df, file.path(res.path, "fea_df.txt"), # 两列，包含算法以及算法所筛选出的变量

sep = "\t", row.names = F, col.names = T, quote = F)

# 对各模型计算C-index

AUC_list <- list()

for (method in methodsValid){

AUC_list[[method]] <- RunEval(fit = model[[method]], # 分类预测模型

Test_set = Test_set, # 测试集预测变量，应当包含训练集中所有的变量，否则会报错

Test_label = Test_class, # 训练集分类数据，应当包含训练集中所有的变量，否则会报错

Train_set = Train_set, # 若需要同时评估训练集，则给出训练集表达谱，否则置NULL

Train_label = Train_class, # 若需要同时评估训练集，则给出训练集分类数据，否则置NULL

Train_name = "TCGA", # 若需要同时评估训练集，可给出训练集的标签，否则按“Training”处理

cohortVar = "Cohort", # 重要：用于指定队列的变量，该列必须存在且指定[默认为“Cohort”]，否则会报错

classVar = classVar) # 用于评估的二元分类变量，必须出现在Test_class中

}

AUC_mat <- do.call(rbind, AUC_list)

write.table(AUC_mat, file.path(res.path, "AUC_mat.txt"),

sep = "\t", row.names = T, col.names = T, quote = F)

# Plot --------------------------------------------------------------------

AUC_mat <- read.table(file.path(res.path, "AUC_mat.txt"),sep = "\t", row.names = 1, header = T,check.names = F,stringsAsFactors = F)

avg_AUC <- apply(AUC_mat, 1, mean) # 计算每种算法在所有队列中平均AUC

avg_AUC <- sort(avg_AUC, decreasing = T) # 对各算法AUC由高到低排序

AUC_mat <- AUC_mat[names(avg_AUC), ] # 对AUC矩阵排序

fea_sel <- fea_list[[rownames(AUC_mat)[1]]] # 最优模型（测试集AUC均值最大）所筛选的特征

avg_AUC <- as.numeric(format(avg_AUC, digits = 3, nsmall = 3)) # 保留三位小数

if(ncol(AUC_mat) < 3) { # 如果用于绘图的队列小于3个

CohortCol <- c("red","blue") # 则给出两个颜色即可（可自行替换颜色）

} else { # 否则通过brewer.pal赋予超过3个队列的颜色

CohortCol <- brewer.pal(n = ncol(AUC_mat), name = "Paired") # 设置队列颜色

}

names(CohortCol) <- colnames(AUC_mat)

cellwidth = 1; cellheight = 0.5

hm <- SimpleHeatmap(AUC_mat, # 主矩阵

avg_AUC, # 侧边柱状图

CohortCol, "steelblue", # 列标签颜色，右侧柱状图颜色

cellwidth = cellwidth, cellheight = cellheight, # 热图每个色块的尺寸

cluster_columns = F, cluster_rows = F) # 是否对行列进行聚类

pdf(file.path(fig.path, "AUC.pdf"), width = cellwidth * ncol(AUC_mat) + 7, height = cellheight * nrow(AUC_mat) * 0.45)

draw(hm)

invisible(dev.off())

# all ML algorithms

RunML <- function(method, Train_set, Train_label, mode = "Model", classVar){

# for example: Enet [alpha=0.4]

method = gsub(" ", "", method) # 去除参数中的空格，得到Enet [alpha=0.4]

method_name = gsub("(\\w+)\\[(.+)\\]", "\\1", method) # get name of ML algorithm, e.g., Enet

method_param = gsub("(\\w+)\\[(.+)\\]", "\\2", method) # get parameter of ML algorithm, e.g., alpha=0.4

method_param = switch(

EXPR = method_name,

"Enet" = list("alpha" = as.numeric(gsub("alpha=", "", method_param))),

"Stepglm" = list("direction" = method_param),

NULL

)

message("Run ", method_name, " algorithm for ", mode, "; ",

method_param, ";",

" using ", ncol(Train_set), " Variables")

args = list("Train_set" = Train_set,

"Train_label" = Train_label,

"mode" = mode,

"classVar" = classVar)

args = c(args, method_param)

obj <- do.call(what = paste0("Run", method_name),

args = args)

if(mode == "Variable"){

message(length(obj), " Variables retained;\n")

}else{message("\n")}

return(obj)

}

RunEnet <- function(Train_set, Train_label, mode, classVar, alpha){

cv.fit = cv.glmnet(x = Train_set,

y = Train_label[[classVar]],

family = "binomial", alpha = alpha, nfolds = 10)

fit = glmnet(x = Train_set,

y = Train_label[[classVar]],

family = "binomial", alpha = alpha, lambda = cv.fit$lambda.min)

fit$subFeature = colnames(Train_set)

if (mode == "Model") return(fit)

if (mode == "Variable") return(ExtractVar(fit))

}

RunLasso <- function(Train_set, Train_label, mode, classVar){

RunEnet(Train_set, Train_label, mode, classVar, alpha = 1)

}

RunRidge <- function(Train_set, Train_label, mode, classVar){

RunEnet(Train_set, Train_label, mode, classVar, alpha = 0)

}

RunStepglm <- function(Train_set, Train_label, mode, classVar, direction){

fit <- step(glm(formula = Train_label[[classVar]] ~ .,

family = "binomial",

data = as.data.frame(Train_set)),

direction = direction, trace = 0)

fit$subFeature = colnames(Train_set)

if (mode == "Model") return(fit)

if (mode == "Variable") return(ExtractVar(fit))

}

RunSVM <- function(Train_set, Train_label, mode, classVar){

data <- as.data.frame(Train_set)

data[[classVar]] <- as.factor(Train_label[[classVar]])

fit = svm(formula = eval(parse(text = paste(classVar, "~."))),

data= data, probability = T)

fit$subFeature = colnames(Train_set)

if (mode == "Model") return(fit)

if (mode == "Variable") return(ExtractVar(fit))

}

RunLDA <- function(Train_set, Train_label, mode, classVar){

data <- as.data.frame(Train_set)

data[[classVar]] <- as.factor(Train_label[[classVar]])

fit = train(eval(parse(text = paste(classVar, "~."))),

data = data,

method="lda",

trControl = trainControl(method = "cv"))

fit$subFeature = colnames(Train_set)

if (mode == "Model") return(fit)

if (mode == "Variable") return(ExtractVar(fit))

}

RunglmBoost <- function(Train_set, Train_label, mode, classVar){

data <- cbind(Train_set, Train_label[classVar])

data[[classVar]] <- as.factor(data[[classVar]])

fit <- glmboost(eval(parse(text = paste(classVar, "~."))),

data = data,

family = Binomial())

cvm <- cvrisk(fit, papply = lapply,

folds = cv(model.weights(fit), type = "kfold"))

fit <- glmboost(eval(parse(text = paste(classVar, "~."))),

data = data,

family = Binomial(),

control = boost_control(mstop = max(mstop(cvm), 40)))

fit$subFeature = colnames(Train_set)

if (mode == "Model") return(fit)

if (mode == "Variable") return(ExtractVar(fit))

}

RunplsRglm <- function(Train_set, Train_label, mode, classVar){

cv.plsRglm.res = cv.plsRglm(formula = Train_label[[classVar]] ~ .,

data = as.data.frame(Train_set),

nt=10, verbose = FALSE)

fit <- plsRglm(Train_label[[classVar]],

as.data.frame(Train_set),

modele = "pls-glm-logistic",

verbose = F, sparse = T)

fit$subFeature = colnames(Train_set)

if (mode == "Model") return(fit)

if (mode == "Variable") return(ExtractVar(fit))

}

RunRF <- function(Train_set, Train_label, mode, classVar){

rf_nodesize = 5 # may modify

Train_label[[classVar]] <- as.factor(Train_label[[classVar]])

fit <- rfsrc(formula = formula(paste0(classVar, "~.")),

data = cbind(Train_set, Train_label[classVar]),

ntree = 1000, nodesize = rf_nodesize,

importance = T,

proximity = T,

forest = T)

fit$subFeature = colnames(Train_set)

if (mode == "Model") return(fit)

if (mode == "Variable") return(ExtractVar(fit))

}

RunGBM <- function(Train_set, Train_label, mode, classVar){

fit <- gbm(formula = Train_label[[classVar]] ~ .,

data = as.data.frame(Train_set),

distribution = 'bernoulli',

n.trees = 10000,

interaction.depth = 3,

n.minobsinnode = 10,

shrinkage = 0.001,

cv.folds = 10,n.cores = 6)

best <- which.min(fit$cv.error)

fit <- gbm(formula = Train_label[[classVar]] ~ .,

data = as.data.frame(Train_set),

distribution = 'bernoulli',

n.trees = best,

interaction.depth = 3,

n.minobsinnode = 10,

shrinkage = 0.001, n.cores = 8)

fit$subFeature = colnames(Train_set)

if (mode == "Model") return(fit)

if (mode == "Variable") return(ExtractVar(fit))

}

RunXGBoost <- function(Train_set, Train_label, mode, classVar){

indexes = createFolds(Train_label[[classVar]], k = 5, list=T)

CV <- unlist(lapply(indexes, function(pt){

dtrain = xgb.DMatrix(data = Train_set[-pt, ],

label = Train_label[-pt, ])

dtest = xgb.DMatrix(data = Train_set[pt, ],

label = Train_label[pt, ])

watchlist <- list(train=dtrain, test=dtest)

bst <- xgb.train(data=dtrain,

max.depth=2, eta=1, nthread = 2, nrounds=10,

watchlist=watchlist,

objective = "binary:logistic", verbose = F)

which.min(bst$evaluation_log$test_logloss)

}))

nround <- as.numeric(names(which.max(table(CV))))

fit <- xgboost(data = Train_set,

label = Train_label[[classVar]],

max.depth = 2, eta = 1, nthread = 2, nrounds = nround,

objective = "binary:logistic", verbose = F)

fit$subFeature = colnames(Train_set)

if (mode == "Model") return(fit)

if (mode == "Variable") return(ExtractVar(fit))

}

RunNaiveBayes <- function(Train_set, Train_label, mode, classVar){

data <- cbind(Train_set, Train_label[classVar])

data[[classVar]] <- as.factor(data[[classVar]])

fit <- naiveBayes(eval(parse(text = paste(classVar, "~."))),

data = data)

fit$subFeature = colnames(Train_set)

if (mode == "Model") return(fit)

if (mode == "Variable") return(ExtractVar(fit))

}

# DRF不适用于二分类情况，因此删去

# RunDRF <- function(Train_set, Train_label, mode, classVar){

# Train_label <- data.frame(

# "0" = as.numeric(Train_label == 0),

# "1" = as.numeric(Train_label == 1)

# )

# fit <- drf(X = Train_set,

# Y = Train_label,

# compute.variable.importance = F)

# fit$subFeature = colnames(Train_set)

#

# summary(predict(fit, functional = "mean", as.matrix(Train_set))$mean)

#

# if (mode == "Model") return(fit)

# if (mode == "Variable") return(ExtractVar(fit))

# }

quiet <- function(..., messages=FALSE, cat=FALSE){

if(!cat){

sink(tempfile())

on.exit(sink())

}

out <- if(messages) eval(...) else suppressMessages(eval(...))

out

}

standarize.fun <- function(indata, centerFlag, scaleFlag) {

scale(indata, center=centerFlag, scale=scaleFlag)

}

# 2.0更新

scaleData <- function(data, cohort = NULL, centerFlags = NULL, scaleFlags = NULL){

samplename = rownames(data)

if (is.null(cohort)){

data <- list(data); names(data) = "training"

}else{

data <- split(as.data.frame(data), cohort)

}

if (is.null(centerFlags)){

centerFlags = F; message("No centerFlags found, set as FALSE")

}

if (length(centerFlags)==1){

centerFlags = rep(centerFlags, length(data)); message("set centerFlags for all cohort as ", unique(centerFlags))

}

if (is.null(names(centerFlags))){

names(centerFlags) <- names(data); message("match centerFlags with cohort by order\n")

}

if (is.null(scaleFlags)){

scaleFlags = F; message("No scaleFlags found, set as FALSE")

}

if (length(scaleFlags)==1){

scaleFlags = rep(scaleFlags, length(data)); message("set scaleFlags for all cohort as ", unique(scaleFlags))

}

if (is.null(names(scaleFlags))){

names(scaleFlags) <- names(data); message("match scaleFlags with cohort by order\n")

}

centerFlags <- centerFlags[names(data)]; scaleFlags <- scaleFlags[names(data)]

outdata <- mapply(standarize.fun, indata = data, centerFlag = centerFlags, scaleFlag = scaleFlags, SIMPLIFY = F)

# lapply(out.data, function(x) summary(apply(x, 2, var)))

outdata <- do.call(rbind, outdata)

outdata <- outdata[samplename, ]

return(outdata)

}

ExtractVar <- function(fit){

Feature <- quiet(switch(

EXPR = class(fit)[1],

"lognet" = rownames(coef(fit))[which(coef(fit)[, 1]!=0)], # 本身没有筛选变量的功能，但是可以舍去模型中系数为0的变量

"glm" = names(coef(fit)), # 逐步回归可以对变量进行筛选

"svm.formula" = fit$subFeature, # SVM对变量没有筛选功能，所以默认使用所有变量

"train" = fit$coefnames, # LDA不能筛选变量，所以默认使用所有变量

"glmboost" = names(coef(fit)[abs(coef(fit))>0]), # glmboost同样不具备筛选变量的功能，因此舍去模型中系数为0的变量

"plsRglmmodel" = rownames(fit$Coeffs)[fit$Coeffs!=0], # plsRglmmodel同样不具备筛选变量的功能，因此舍去模型中系数为0的变量

"rfsrc" = var.select(fit, verbose = F)$topvars, # rfsrc可以对变量进行筛选

"gbm" = rownames(summary.gbm(fit, plotit = F))[summary.gbm(fit, plotit = F)$rel.inf>0], # gbm通过舍去重要性为0的变量来进行变量筛选

"xgb.Booster" = fit$subFeature, # xgboost没有筛选变量的能力， 默认使用所有变量

"naiveBayes" = fit$subFeature # naiveBayes没有筛选变量的能力，默认使用所有变量

# "drf" = fit$subFeature # drf自带的变量系数提取函数会输出NA，因此默认使用所有变量

))

Feature <- setdiff(Feature, c("(Intercept)", "Intercept"))

return(Feature)

}

CalPredictScore <- function(fit, new_data, type = "lp"){

new_data <- new_data[, fit$subFeature]

RS <- quiet(switch(

EXPR = class(fit)[1],

"lognet" = predict(fit, type = 'response', as.matrix(new_data)), # response

"glm" = predict(fit, type = 'response', as.data.frame(new_data)), # response

"svm.formula" = predict(fit, as.data.frame(new_data), probability = T), #

"train" = predict(fit, new_data, type = "prob")[[2]],

"glmboost" = predict(fit, type = "response", as.data.frame(new_data)), # response

"plsRglmmodel" = predict(fit, type = "response", as.data.frame(new_data)), # response

"rfsrc" = predict(fit, as.data.frame(new_data))$predicted[, "1"],

"gbm" = predict(fit, type = 'response', as.data.frame(new_data)), # response

"xgb.Booster" = predict(fit, as.matrix(new_data)),

"naiveBayes" = predict(object = fit, type = "raw", newdata = new_data)[, "1"]

# "drf" = predict(fit, functional = "mean", as.matrix(new_data))$mean

))

RS = as.numeric(as.vector(RS))

names(RS) = rownames(new_data)

return(RS)

}

PredictClass <- function(fit, new_data){

new_data <- new_data[, fit$subFeature]

label <- quiet(switch(

EXPR = class(fit)[1],

"lognet" = predict(fit, type = 'class', as.matrix(new_data)),

"glm" = ifelse(test = predict(fit, type = 'response', as.data.frame(new_data))>0.5,

yes = "1", no = "0"), # glm不返回预测的类，将概率>0.5的作为1类

"svm.formula" = predict(fit, as.data.frame(new_data), decision.values = T), #

"train" = predict(fit, new_data, type = "raw"),

"glmboost" = predict(fit, type = "class", as.data.frame(new_data)),

"plsRglmmodel" = ifelse(test = predict(fit, type = 'response', as.data.frame(new_data))>0.5,

yes = "1", no = "0"), # plsRglm不允许使用因子变量作为因变量，因而predict即使type设为class也无法正常运作

"rfsrc" = predict(fit, as.data.frame(new_data))$class,

"gbm" = ifelse(test = predict(fit, type = 'response', as.data.frame(new_data))>0.5,

yes = "1", no = "0"), # gbm未设置预测类别，设置大于0.5为1

"xgb.Booster" = ifelse(test = predict(fit, as.matrix(new_data))>0.5,

yes = "1", no = "0"), # xgboost 未提供预测类别，设置大于0.5为1

"naiveBayes" = predict(object = fit, type = "class", newdata = new_data)

# "drf" = predict(fit, functional = "mean", as.matrix(new_data))$mean

))

label = as.character(as.vector(label))

names(label) = rownames(new_data)

return(label)

}

RunEval <- function(fit,

Test_set = NULL,

Test_label = NULL,

Train_set = NULL,

Train_label = NULL,

Train_name = NULL,

cohortVar = "Cohort",

classVar){

if(!is.element(cohortVar, colnames(Test_label))) {

stop(paste0("There is no [", cohortVar, "] indicator, please fill in one more column!"))

}

if((!is.null(Train_set)) & (!is.null(Train_label))) {

new_data <- rbind.data.frame(Train_set[, fit$subFeature],

Test_set[, fit$subFeature])

if(!is.null(Train_name)) {

Train_label$Cohort <- Train_name

} else {

Train_label$Cohort <- "Training"

}

colnames(Train_label)[ncol(Train_label)] <- cohortVar

Test_label <- rbind.data.frame(Train_label[,c(cohortVar, classVar)],

Test_label[,c(cohortVar, classVar)])

Test_label[,1] <- factor(Test_label[,1],

levels = c(unique(Train_label[,cohortVar]), setdiff(unique(Test_label[,cohortVar]),unique(Train_label[,cohortVar]))))

} else {

new_data <- Test_set[, fit$subFeature]

}

RS <- suppressWarnings(CalPredictScore(fit = fit, new_data = new_data))

Predict.out <- Test_label

Predict.out$RS <- as.vector(RS)

Predict.out <- split(x = Predict.out, f = Predict.out[,cohortVar])

unlist(lapply(Predict.out, function(data){

as.numeric(auc(suppressMessages(roc(data[[classVar]], data$RS))))

}))

}

SimpleHeatmap <- function(Cindex_mat, avg_Cindex,

CohortCol, barCol,

cellwidth = 1, cellheight = 0.5,

cluster_columns, cluster_rows){

col_ha = columnAnnotation("Cohort" = colnames(Cindex_mat),

col = list("Cohort" = CohortCol),

show_annotation_name = F)

row_ha = rowAnnotation(bar = anno_barplot(avg_Cindex, bar_width = 0.8, border = FALSE,

gp = gpar(fill = barCol, col = NA),

add_numbers = T, numbers_offset = unit(-10, "mm"),

axis_param = list("labels_rot" = 0),

numbers_gp = gpar(fontsize = 9, col = "white"),

width = unit(3, "cm")),

show_annotation_name = F)

Heatmap(as.matrix(Cindex_mat), name = "AUC",

right_annotation = row_ha,

top_annotation = col_ha,

# col = c("#1CB8B2", "#FFFFFF", "#EEB849"), # 黄绿配色

col = c("#4195C1", "#FFFFFF", "#CB5746"), # 红蓝配色

rect_gp = gpar(col = "black", lwd = 1), # 边框设置为黑色

cluster_columns = cluster_columns, cluster_rows = cluster_rows, # 不进行聚类，无意义

show_column_names = FALSE,

show_row_names = TRUE,

row_names_side = "left",

width = unit(cellwidth * ncol(Cindex_mat) + 2, "cm"),

height = unit(cellheight * nrow(Cindex_mat), "cm"),

column_split = factor(colnames(Cindex_mat), levels = colnames(Cindex_mat)),

column_title = NULL,

cell_fun = function(j, i, x, y, w, h, col) { # add text to each grid

grid.text(label = format(Cindex_mat[i, j], digits = 3, nsmall = 3),

x, y, gp = gpar(fontsize = 10))

}

)

}
